# Evaluating the Role of Robotic Surgery Gastric Cancer Treatment: A Comprehensive Review by the Robotic Global Surgical Society (TROGSS) and European Federation International Society for Digestive Surgery (EFISDS) Joint Working Group

**DOI:** 10.3390/curroncol32020083

**Published:** 2025-01-31

**Authors:** Luigi Marano, Tomasz Cwalinski, Sergii Girnyi, Jaroslaw Skokowski, Aman Goyal, Silvia Malerba, Francesco Paolo Prete, Piotr Mocarski, Magdalena Kamila Kania, Maciej Świerblewski, Marek Strzemski, Luis Osvaldo Suárez-Carreón, Johnn Henry Herrera Kok, Karol Polom, Witold Kycler, Valentin Calu, Pasquale Talento, Antonio Brillantino, Francesco Antonio Ciarleglio, Luigi Brusciano, Nicola Cillara, Ruslan Duka, Beniamino Pascotto, Juan Santiago Azagra, Natale Calomino, Mario Testini, Adel Abou-Mrad, Rodolfo J. Oviedo, Yogesh Vashist

**Affiliations:** 1 Department of Medicine, Academy of Applied Medical and Social Sciences—AMiSNS (Akademia Medycznych I Spolecznych Nauk Stosowanych), 52-300 Elbląg, Poland; j.skokowski@amisns.edu.pl (J.S.); s.malerba10@studenti.uniba.it (S.M.); k.polom@amisns.edu.pl (K.P.); 2 Department of General Surgery and Surgical Oncology, “Saint Wojciech” Hospital, “Nicolaus Copernicus” Health Center, 80-000 Gdańsk, Poland; tcwalinski@copernicus.gda.pl (T.C.); sgirnyi@copernicus.gda.pl (S.G.); pmocarski@copernicus.gda.pl (P.M.); mkania@copernicus.gda.pl (M.K.K.); mswierblewski@copernicus.gda.pl (M.Ś.); 3 Department of Surgery, Dnipro State Medical University, Volodymyra Vernadskoho St. 9, 49044 Dnipro, Ukraine; rusduka@gmail.com; 4 Department of Medicine, Surgery, and Neurosciences, University of Siena, 53100 Siena, Italy; natale.calomino@unisi.it; 5 Department of General Surgery, Mahatma Gandhi Medical College, Research Institute, Pondicherry, Cuddalore Rd., ECR, Pillayarkuppam 607402, Puducherry, India; doc.aman.goyal@gmail.com; 6 Department of Medicine, Adesh Institute of Medical Sciences and Research, Bathinda 151001, Punjab, India; 7 Department of Precision and Regenerative Medicine and Ionian Area, University of Bari “Aldo Moro”, 70110 Bari, Italy; francesco.prete@uniba.it (F.P.P.); mario.testini@uniba.it (M.T.); 8 Department of Anesthesiology and Intensive Care, “Saint Wojciech” Hospital, “Nicolaus Copernicus” Health Center, 80-000 Gdańsk, Poland; mstrzemski@copernicus.gda.pl; 9 Department of Bariatric Surgery, UMAE Hospital de Especialidades del Centro Medico Nacional de Occidente, Guadalajara 44349, Mexico; osvaldo.suarez@academico.udg.mx; 10 Department of Surgery, Universidad de Guadalajara, Guadalajara 44340, Mexico; 11 Department of Surgery, Complejo Asistencial Universitario de Palencia, 34401 Palencia, Spain; jherrerak@saludcastillayleon.es; 12 Department of Gastrointestinal Surgical Oncology, Greater Poland Cancer Centre, 61-866 Poznan, Poland; witold.kycler@wco.pl; 13 Department of Surgery, University of Medicine and Pharmacy Carol Davila, 010001 Bucharest, Romania; valentin.calu@umfcd.ro; 14 Department of Surgery, Pelvic Floor Center, AUSL-IRCCS Reggio Emilia, 42122 Reggio Emilia, Italy; pasquale.talento@ausl.re.it; 15 Department of Surgery, Antonio Cardarelli Hospital, 80131 Naples, Italy; antonio.brillantino@aocardarelli.it; 16 Department of General Surgery, Hepato-Pancreato-Biliary (HPB) Unit—APSS, 38121 Trento, Italy; francesco.ciarleglio@apss.tn.it; 17 Division of General, Oncological, Mini-Invasive and Obesity Surgery, University of Study of Campania “Luigi Vanvitelli”, 80131 Naples, Italy; luigi.brusciano@unicampania.it; 18 Department of Surgery, “SS. Trinità” Hospital, 09121 Cagliari, Italy; 19 Department of General and Minimally Invasive Surgery (Laparoscopy & Robotic), Centre Hospitalier de Luxembourg, 1210 Luxembourg, Luxembourg; pascotto.b@ch.lu (B.P.); azagra.js@chl.lu (J.S.A.); 20 Department of Surgery, Centre Hospitalier Universitaire d’Orléans, 45000 Orléans, France; adel.abou-mrad@orange.fr; 21 Department of Surgery, Nacogdoches Medical Center, Nacogdoches, TX 75962, USA; 22 Department of Surgery, Tilman J. Fertitta Family College of Medicine, University of Houston, Houston, TX 77001, USA; 23 Department of Surgery, Sam Houston State University College of Osteopathic Medicine, Conroe, TX 77301, USA; 24 Department of Surgery, Organ Transplant Center for Excellence, Center for Liver Diseases and Oncology, King Faisal Specialist Hospital and Research Center, Riyadh 12271, Saudi Arabia; y.vashist@kfu.edu.sa

**Keywords:** robot-assisted gastrectomy, minimally invasive surgery, gastric cancer surgery, learning curve, lymphadenectomy

## Abstract

Introduction: Robot-assisted minimally invasive gastrectomy (RAMIG) represents a significant advancement in the surgical management of gastric cancer, offering superior dexterity, enhanced visualization, and improved ergonomics compared to laparoscopic gastrectomy (LG). This review systematically evaluates the current evidence on perioperative outcomes, oncological efficacy, learning curves, and economic considerations, providing insights into RAMIG’s potential role in modern gastric cancer surgery. Methods: A thorough analysis of retrospective, prospective, and meta-analytic studies was conducted to compare RAMIG with LG. Key outcomes, including operative time, intraoperative blood loss, lymph node retrieval, postoperative complications, learning curve duration, and cost-effectiveness, were assessed. Emphasis was placed on both short-term and long-term oncological outcomes to determine the clinical value of RAMIG. Results: Evidence indicates that RAMIG is associated with reduced intraoperative blood loss, lower morbidity rates, and a shorter learning curve, with proficiency achieved after 11–25 cases compared to 40–60 cases for LG. The robotic platform’s articulated instruments and enhanced three-dimensional visualization enable more precise lymphadenectomy, particularly in complex anatomical regions. Despite these advantages, operative time remains longer, and costs remain higher due to system acquisition, maintenance, and consumable expenses. However, emerging data suggest a gradual narrowing of cost disparities. While short-term outcomes are favorable, further high-quality, multicenter studies are needed to validate long-term oncological efficacy and survival outcomes. Conclusion: RAMIG offers significant technical and clinical advantages over conventional LG, particularly in terms of precision and learning efficiency. However, the long-term oncological benefits and economic feasibility require further validation. Future research should focus on cost optimization, advanced technological integration such as near-infrared fluorescence and artificial intelligence, and multicenter trials to solidify RAMIG’s role as a standard approach for gastric cancer surgery.

## 1. Introduction

Minimally invasive techniques for gastric cancer surgery have been increasingly utilized to enhance postoperative recovery for patients undergoing gastrectomy [1]. These approaches have demonstrated benefits such as reduced postoperative pain, decreased complication rates, minimal blood loss, shorter durations of hospitalization, and quicker resumption of daily activities [2]. Since its introduction in the late 1990s, robotic surgery has gained widespread adoption, with significant advancements and growing expertise over time [3,4]. Notably, robotic systems have addressed several limitations inherent to conventional laparoscopy by offering enhanced precision through tremor filtration, articulated instruments with wrist-like motion, seven degrees of freedom, and motion scaling capabilities [5,6].

Numerous proficient laparoscopic surgeons have adopted robotic surgery techniques for the treatment of gastric cancer. Within a decade of its initial application for early-stage gastric cancer, robotic gastrectomy has emerged as a safe and viable alternative to traditional laparoscopic approaches [7,8]. An umbrella review of systematic reviews and meta-analyses reported that robot-assisted minimally invasive gastrectomy (RAMIG) is associated with reduced intraoperative blood loss (weighted mean difference [WMD] −29.86 mL; 95% CI:−46.24 to −13.47; *p* < 0.001) and shorter time to oral intake (WMD −0.28 days; 95% CI: −0.46 to −0.09; *p* < 0.01), while requiring a longer operation time (WMD +57.15 min; 95% CI: +42.26 to +72.05; *p* < 0.001) [6]. Hospital stay was marginally shorter in the robotic group, though not reaching consistent statistical significance across studies. Additionally, robotic surgery demonstrated equivalent safety profiles with no significant differences in total complication rates, mortality, or morbidity compared to laparoscopic approaches [6]. These findings highlight the potential of RAMIG to improve perioperative outcomes, particularly in complex gastric cancer surgeries, while maintaining comparable safety and oncological efficacy. However, despite its advantages, challenges such as high costs and the need to establish its oncological efficacy for advanced gastric cancer remain significant hurdles to broader adoption [9]. This review, conducted by a joint working group on behalf of the Robotic Global Surgical Society (TROGSS) and the European Federation of the International Society for Digestive Surgery (EFISDS), aims to provide an in-depth analysis of the current evidence surrounding robotic gastrectomy, including its clinical applications, perioperative outcomes, cost considerations, learning curve, oncological outcomes, and potential future developments. This study represents the largest review of its kind in the literature to date on RAMIG in comparison to laparoscopic gastrectomy (LG). It analyzes safety outcomes in depth and includes a discussion on multidisciplinary management as well as suggestions for the advancement of robotics in the field of minimally invasive gastrectomy for cancer.

## 2. Materials and Methods

We conducted a comprehensive literature search across several online databases, including the Cochrane Library, Embase, PubMed, and Web of Science, to identify studies on RAMIG published up to June 2024. The search strategy employed a combination of subject headings and text words, incorporating terms such as “robotic” “gastrectomy,” and their synonyms to ensure comprehensive coverage and minimize the chance of missing relevant studies. Boolean operators (“AND” and “OR”) were used to refine the search. We included studies that compared RAMIG to conventional approaches, such as laparoscopic or open surgery, for the treatment of both cardia and noncardia gastric cancer. Additionally, noncomparative studies and those focusing solely on robotic techniques without comparisons to conventional surgery were also included. Eligibility criteria required studies to be written in English and to report data on more than 10 patients. Articles such as narrative reviews, study protocols, invited commentaries, studies without full-text availability, and duplicates were excluded (Table 1). This process ensured a robust selection of relevant literature while maintaining a comprehensive scope.

## 3. Clinical Applications, Perioperative Outcomes, and Emerging Technologies

### 3.1. Evolving Indications and Applications of Robotic Gastrectomy in Gastric Cancer Management

The application of RAMIG has undergone significant evolution, paralleling advancements in LG for the management of gastric cancer. Initially, RAMIG was primarily indicated for early-stage gastric cancer in patients without clinical evidence of lymph node metastasis. Over time, its indications have broadened to include clinical-stage T1–T2 tumors, irrespective of perigastric lymph node involvement, except for lesions suitable for endoscopic submucosal dissection (ESD) [10,11]. This expansion reflects technological advancements and an increasing body of clinical expertise. Robot-assisted surgery has become an integral part of clinical practice for gastric cancer (GC) worldwide, mirroring its adoption in other fields of abdominal and pelvic surgery. RAMIG aims to provide benefits comparable to those of LG while addressing ergonomic limitations inherent to conventional laparoscopy. Key features of robotic systems include high-resolution, three-dimensional imaging with a surgeon-controlled stable camera, tremor suppression, and articulated instruments with greater degrees of freedom, all of which contribute to an optimized surgical environment. However, the significantly higher procedural cost remains a substantial barrier to widespread adoption [12,13,14]. The integration of minimally invasive techniques, including RAMIG and LG, for advanced GC involving serosal invasion, has been limited, particularly in regions like Korea and Japan. Nonetheless, emerging data suggest that serosal involvement may not represent an absolute contraindication for minimally invasive surgical approaches. Despite these advancements, specific limitations persist, particularly in scenarios involving large tumors, extensive lymphadenopathy, or cases requiring multi-organ resection, which can constrain the feasibility of these techniques [7,15]. Achieving an R0 resection, a critical surgical objective, necessitates the precise determination of the proximal resection margin. For tumors that are either small or nonpalpable, methods such as preoperative endoscopic placement of radiopaque hemoclips or intraoperative endoscopic localization have been advocated to facilitate accurate tumor localization [16,17,18,19].

The scope of lymphadenectomy in RAMIG adheres to the guidelines outlined in the Japanese Classification of Gastric Carcinoma. For clinically early-stage gastric cancer without lymph node metastasis, a D1+ lymphadenectomy is recommended, whereas D2 dissection is advised for advanced gastric cancer or cases with confirmed regional lymph node involvement [15]. Notably, recent evidence indicates that RAMIG achieves lymph node retrieval rates comparable to, or even exceeding, those of LG, particularly in anatomically complex regions such as the splenic hilum and the suprapancreatic area [9]. Asian surgeons have led the exploration of RAMIG’s feasibility, safety, and efficacy. In 2021, two randomized controlled trials (RCTs) comparing RAMIG to LG demonstrated lower postoperative morbidity, faster recovery, and a higher lymph node yield with RAMIG in one of the trials [13,14]. These findings were corroborated by a large propensity score-matched cohort study involving over 3500 patients [12]. Despite these promising results, concerns about prolonged operative time and high costs remain. Ongoing research is focused on refining surgical techniques, including resection, reconstruction, anastomosis, and lymphadenectomy [20]. A Japanese phase III RCT (JCOG1907, MONA LISA study) is currently evaluating whether RAMIG can achieve superior outcomes compared to LG, particularly in reducing postoperative complications [21]. As experience with RAMIG grows, further advancements are expected. However, it remains uncertain whether these improvements will translate into better long-term survival outcomes for patients.

### 3.2. Evolution of Minimally Invasive Techniques in Robotic Gastrectomy

Since the first laparoscopic distal gastrectomy (LDG) was performed by Kitano in 1991 and described in 1994 [22], a minimally invasive approach has been accepted as the gold standard for distal gastrectomies [23,24,25,26,27].

The evidence is different for total gastrectomy because of the technical difficulties of the procedure. Azagra et al. first described laparoscopic total gastrectomy (LTG) for cancer in 1993 [28]. Since then, many authors described the feasibility and safety of the LTG for early and, recently, advanced gastric cancer [8,9,10,11,12,13,14,15,16,17,18,29,30,31,32,33,34,35,36]. The limited adoption of LTG primarily stems from two factors: achieving adequate lymphadenectomy and performing esophagojejunostomy (EJ). The first one impacts oncologic safety and the second one impacts surgical safety. Both impact the patient’s prognosis [37,38,39,40,41,42,43,44].

Various techniques of EJ construction have been described, including mechanics and hand-sewn (HS). Mechanical methods use the circular stapler (CS) and the linear stapler (LS). Up to now there is no standard on superiority and no consensus for EJ construction [41,45,46,47,48].

LTG most commonly is a laparoscopic-assisted total gastrectomy (LATG) because the EJ is performed using a CS by a minilaparotomy [49,50,51,52,53,54].

While this approach is widely practiced, the minilaparotomy and manipulation required for CS EJ can increase postoperative pain and anastomotic bleeding [50]. LS EJ, facilitated by laparoscopic techniques, allows for intracorporeal anastomosis and eliminates the need for a minilaparotomy, contributing to cost savings by reusing stapler cartridges [37,40,48,55]. However, LS EJ is limited in constructing higher anastomoses due to the need for significant esophageal stump dissection, which may compromise vascularization [37,40,48,55].

RAMIG has shown significant potential in addressing these limitations, particularly for HS EJ. HS EJ has long been recognized for its theoretical advantages, including improved blood supply, reduced tension on the sutures, and the ability to perform high esophageal transections when needed [42,43,44,56,57,58,59,60]. Despite these benefits, its adoption has been limited by the technical difficulty of intracorporeal suturing. Robotic platforms, with their enhanced visualization, tremor-free precision, and wristed instruments, make HS EJ significantly more feasible and reproducible. The implementation of standardized techniques, such as Azagra’s approach using barbed sutures, has further simplified the procedure and demonstrated excellent outcomes with minimal anastomosis-related complications [56,58,59]. The precise control offered by robotic systems provides surgeons with a greater sense of safety, as every suture is placed under optimal visualization and minimal manipulation of the anastomosis.

EJ-related complications, including leakage and stenosis, remain critical challenges, with reported rates ranging from 2% to 14% [61,62,63,64,65]. Notably, the KLASS-03 trial for laparoscopic total gastrectomy reported a 3.2% incidence of anastomosis-related complications [66], while a Japanese nationwide surgical registry reported 4.4% after open TG [67]. EJ leakage rates specifically range from 1.7% to 15%, with an average incidence of 4.4% and associated mortality rates up to 30% [61,63,67,68,69,70]. The Italian Research Group for Gastric Cancer (GIRCG) reported an EJ leakage rate of 6.6%, with a mortality rate of 8.6% [46]. These findings underscore the complexity of EJ after minimally invasive total gastrectomy and highlight the potential of RAMIG to mitigate these risks.

Robotic platforms overcome many of the challenges associated with mechanical EJ techniques. CS EJ is particularly dependent on the assistant’s skill for circular stapler manipulation, which can be restricted by limited access due to robotic arms [49,54,71,72,73,74,75,76,77]. LS EJ, while advantageous in many laparoscopic settings, is constrained by poor visualization of high esophageal stump anastomoses and increased tension at the anastomosis site. In contrast, robotic platforms allow for improved intracorporeal HS EJ with minimal esophageal stump mobilization, reducing tension and improving outcomes [41,42,43,44,57,60,78,79,80,81,82,83]. The introduction of barbed sutures in robotic HS EJ has further reduced operative time and technical complexity, with outcomes comparable to or superior to nonbarbed HS techniques [41,42,44,57,84,85,86,87,88,89,90,91,92,93,94].

The advantages of RAMIG extend beyond suturing. Robotic platforms enhance lymphadenectomy by providing superior precision in complex dissections. This capability, combined with the reproducibility of HS EJ, positions RAMIG as a promising standard for minimally invasive total gastrectomy. While HS EJ requires a significant learning curve [41,81,82,84,85], the integration of robotic technology has the potential to overcome this barrier, facilitating broader adoption. Ultimately, robotic HS EJ may become the gold standard for alimentary tract reconstruction after total gastrectomy, offering improved oncologic and surgical outcomes.

### 3.3. Standard Surgical Steps in RAMIG

The patient is positioned supine on a split operating table, with a nasogastric tube placed for gastric decompression. The pneumoperitoneum is established in the left upper quadrant using a Veress needle, maintaining an intra-abdominal pressure of 13 mmHg. A robotic port for 30° optics is placed laterally to the umbilicus. Under direct visualization, three additional robotic trocars (8 mm) are inserted: two at the midclavicular line in the upper abdomen and one at the right anterior axillary line for liver retraction. Following trocar placement, the patient is tilted into a reverse Trendelenburg position at an angle of 15–20°. The robotic platform, typically the Xi Da Vinci Surgical System, is docked at the head of the operating table, and the robotic arms are attached to the ports. A 12-mm assistant port is positioned between the left robotic port and the camera port for the introduction of auxiliary instruments, including aspiration devices, clip appliers, sutures, and staplers [41,42,44,57,84,85,86,87,88,89,90,91,92,93,94].

#### 3.3.1. Robot-Assisted Subtotal Gastrectomy (RASG)

The procedure begins with the division of the greater omentum, proceeding from the gastric body toward the pylorus and the lower pole of the spleen. This dissection is performed using monopolar cautery or bipolar forceps. In patients with high adiposity, an advanced energy device, such as a robotic ultrasound dissector, may be utilized to enhance surgical precision and efficiency. The dissection continues to the short gastric vessels, which are ligated at their roots, encompassing lymph node stations 4sb and 4d [41,81,82,84,85].

The right gastroepiploic vessels are dissected en bloc with lymphatic tissue, using anatomical landmarks such as Henle’s trunk and the inferior pancreatic border to identify and ligate the right gastroepiploic vein and artery. This step also facilitates the en bloc removal of lymph node station 6. The lesser omentum is then opened from the pars flaccida to the hepatic pedicle to allow for the dissection of lymph node stations 3 and 5. The duodenum is mobilized and transected, enabling the removal of lymph node stations 12a and 8a. Lymphadenectomy is extended toward the celiac trunk, exposing and ligating the left gastric artery and vein and excising station 7 lymph nodes. Depending on tumor characteristics, dissection may also involve lymph nodes along the splenic artery (stations 10 and 11p) [42,43,44,45,46,47,48,49,50,51,80,81,82,83]. The stomach is subsequently transected at the proximal third using an articulated linear stapler, and the resected specimen, including the stomach, omentum, and lymphatic tissue, is retrieved via a small pararectal incision. Reconstruction is achieved via a Roux-en-Y gastrojejunostomy, typically performed intracorporeally. The gastrojejunostomy is performed using a side-to-side stapled technique, with the entry site closed by absorbable sutures. Alternatively, an end-to-side, robot-sewn anastomosis is constructed using continuous absorbable sutures for the posterior and anterior layers to ensure secure and tension-free anastomosis [41,42,44,57,84,85,86,87,88,89,90,91,92,93,94].

#### 3.3.2. Robot-Assisted Total Gastrectomy (RATG)

RATG follows similar surgical principles to RASG, with specific modifications for the dissection and reconstruction phases. The gastrosplenic ligament is divided to mobilize the upper portion of the greater curvature and to remove lymph nodes at stations 4sa and 2. D2 lymphadenectomy involves the removal of lymph nodes along the splenic artery (stations 11d and 10), with spleen preservation unless directly invaded or affected by metastasis [49,54,71,72,73,74,75,76,77]. The abdominal esophagus is mobilized and transected using a linear stapler, ensuring an adequate proximal margin for oncological safety. Reconstruction is performed via an intracorporeal Roux-en-Y esophagojejunostomy. This can be executed with a circular stapler or a robot-assisted, hand-sewn technique. For the stapler method, a robotic purse-string suture is placed on the esophageal stump to secure the anvil, which is then connected to the stapler introduced via a pararectal incision. The jejunal stump is closed with a linear stapler. In the hand-sewn approach, continuous absorbable sutures are used to construct both the posterior and anterior layers of the anastomosis [41,42,44,57,84,85,86,87,88,89,90,91,92,93,94]. The reconstruction phase is completed with a jejunojejunostomy positioned 40 cm distal to the esophagojejunostomy.

#### 3.3.3. Robotic D2 Lymphadenectomy

Robotic D2 lymphadenectomy is a technically demanding but critical component of gastrectomy for gastric cancer, requiring precise dissection of lymphatic tissue around major vascular structures [61,63,67,68,69,70]. The procedure begins with dissection along the hepatic pedicle, continuing to the common hepatic artery and celiac trunk. Lymph nodes at stations 12a, 8a, and 7 are removed en bloc. The left gastric artery is identified and ligated at its origin, enabling dissection of station 9 lymph nodes.

Robotic platforms provide distinct advantages in this phase, particularly in the dissection of the suprapancreatic region and along the splenic artery (station 11p) [46]. The stereoscopic imaging and articulation of robotic instruments allow surgeons to access deep, narrow spaces with greater precision, minimizing the risk of vascular injury. The convex contour of the pancreas, often a challenge in laparoscopy, is navigated with ease using the robotic system. Dissection along the splenic artery ensures the preservation of vascular branches to the pancreas and spleen, facilitating a spleen-preserving D2 lymphadenectomy [46].

Additionally, robotic systems offer superior control during unexpected vascular bleeding, a common occurrence in the infrapyloric (station 6) and suprapancreatic regions (stations 7–9). The surgeon’s ability to use three robotic instruments simultaneously for clamping, suturing, and achieving hemostasis significantly enhances intraoperative safety. These technical advantages position robotic D2 lymphadenectomy as a highly effective approach for achieving oncological clearance while minimizing complications [61,62,63,64,65].

### 3.4. Perioperative Outcomes

#### 3.4.1. Operative Time

Numerous studies, including randomized controlled trials (RCTs), have consistently demonstrated that RAMIG typically requires longer operative times compared to LG. Reported durations for RAMIG range from 202 to 439 min, while LG durations vary between 171 and 361 min [7,95,96]. Specific RCTs have confirmed this difference, with Wang G et al. reporting mean durations of 242.7 min for RAMIG versus 192.4 min for LG (*p* = 0.002), Lu et al. documenting 201.2 min for RAMIG compared to 181.6 min for LG (*p* < 0.001), and Ojima T et al. noting 297 min for RAMIG versus 245 min for LG (*p* = 0.001) [13,14,97]. Similarly, a nonrandomized prospective study by Kim H et al. found RAMIG to take significantly longer than LG, with durations of 221 and 178 min, respectively (*p* < 0.001) [98]. Other prospective studies have reported RAMIG durations ranging from 313 to 372 min [99,100,101]. Retrospective studies, both multi-institutional and single-center, have also demonstrated longer operative times for RAMIG, with differences typically ranging from 20 to 50 min compared to LG [12,102,103,104,105,106,107].

An umbrella review of 14 systematic reviews and meta-analyses further confirmed that RAMIG involves longer operative times compared to LG, encompassing 146 primary studies and over 37,500 patients [9]. Statistically significant differences in operative duration were observed in eleven studies [6,108,109,110,111,112,113,114,115,116,117]. This prolonged operative time has been attributed to additional steps, such as robotic docking and undocking, as reported in the literature [118]. However, the evidence supporting this association is relatively weak. Liu et al. analyzed contributing factors and found that while the effective operative time and frequency of instrument exchanges were similar between RAMIG and LG, “junk time”, including robotic arm setup and positioning, was significantly longer for RAMIG [119].

Despite advancements in surgical expertise and familiarity with robotic systems, extended operative time remains a notable limitation of RAMIG. A prior meta-analysis confirmed a mean operative time of 267.34 min for RAMIG compared to 220.48 min for LG (*p* < 0.001) [120]. Conflicting findings do exist; Pan et al. reported no significant differences in operative times between RAMIG and LG [121], while Omori T et al. recently indicated that RAMIG could achieve shorter operative times than LG through training and accumulated expertise [122].

#### 3.4.2. Blood Loss

Blood loss during RAMIG has been extensively studied, with varying results across investigations. Several analyses have demonstrated a significant reduction in intraoperative blood loss with RAMIG compared to LG, with estimates ranging from 46 to 176 mL for RAMIG and 34 to 212 mL for LG [5,95,96]. Furthermore, three RCTs reported significantly lower intraoperative blood loss during RAMIG compared to LG or open gastrectomy (OG). Wang G et al. documented mean blood loss of 94.2 mL for RAMIG versus 152.8 mL for LG, Pan HF et al. observed 41.3 mL for RAMIG compared to 83.7 mL for LG, and Lu J et al. reported 41.2 mL for RAMIG versus 55.7 mL for LG [14,97,121]. However, some studies have reported conflicting findings. An RCT by Ojima T et al. found no significant difference in blood loss between RAMIG and LG (25 mL for both, *p* = 0.18) [13], while a nonrandomized prospective study by Kim H et al. also found similar results (50 mL vs. 55 mL, *p* = 0.318) [98]. Despite these discrepancies, most studies report a reduction in intraoperative bleeding with RAMIG, with only three studies failing to show statistical significance in this difference [105,123,124]. This reduction may be attributed to the enhanced visualization provided by the robotic 3D optical system, combined with superior precision in fine movements and tremor-filtering capabilities [125].

Prospective studies from Japan have further highlighted minimal intraoperative blood loss during RAMIG, with estimates ranging from 15 to 20 mL [99,100,101]. Retrospective studies have produced more variable outcomes. For instance, Li et al. observed significantly lower blood loss with RAMIG compared to LG (126.8 vs. 142.5 mL, *p* < 0.0001) [12], whereas another study indicated no significant differences (20 vs. 15 mL, *p* = 0.149) [102]. Among seven single-center retrospective studies, four demonstrated the superiority of RAMIG in reducing intraoperative blood loss [105,106,107,122], while two reported no significant differences between RAMIG and LG [104,126]. Interestingly, one study observed slightly higher blood loss with RAMIG compared to LG (37 vs. 28 mL, *p* = 0.005) [103]; however, the small volumes in both groups limit the practical significance of these findings. Across most studies, the difference in estimated intraoperative blood loss between RAMIG and LG was approximately 20 mL.

Meta-analyses provide further clarity, showing significantly lower blood loss during RAMIG compared to LG (98.77 vs. 115.02 mL, *p* < 0.001) [120]. This reduction is largely attributed to the technological innovations of RAMIG, including high-resolution 3D visualization and tremor-filtered, articulated instruments, which enhance vascular identification and control of intra-abdominal bleeding. Although the short-term clinical significance of reduced blood loss may be minimal, its potential impact on long-term oncological outcomes, particularly in advanced gastric cancer, remains an important area of research [102,127].

#### 3.4.3. Conversion to Open Surgery

Among all studies analyzing the rate of conversion from robotic and laparoscopic approaches to open surgery, almost all authors consistently reported relatively low conversion rates for both techniques [1,13,103,111,112,117,120]. For instance, Ojima et al. observed conversion rates of 3.4% for the robotic approach and 1.6% for the laparoscopic approach [13]. However, no statistically significant differences were found across these studies. Additionally, a recent umbrella review highlighted a higher risk of conversion to open surgery in robotic gastrectomy, as reported by all three studies investigating this outcome [9,108,111]. Despite these findings, no statistically significant differences were observed. These results suggest that while conversion is a relevant consideration in minimally invasive gastrectomy, both approaches demonstrate comparable and acceptable conversion rates.

#### 3.4.4. Morbidity

To reliably evaluate morbidity, only complications classified as Clavien–Dindo (CD) grade ≥ IIIa were included in most analyses, as these events are potentially life-threatening and often require surgical, endoscopic, or radiological interventions. Such complications can lead to prolonged hospital stays and increased healthcare costs [128,129]. While many studies have reported similar overall complication rates between RAMIG and LG, the specific findings vary. A recent multicenter prospective study observed complication rates of 11.9% for RAMIG and 10.3% for LG, with major complications (CD grade ≥ IIIa) occurring at a rate of 1.1% in both groups [98]. Conversely, Ojima T et al. reported significantly fewer overall complications with RAMIG compared to LG (5.3% vs. 16.2%, *p* = 0.01), although no significant differences were observed for intra-abdominal infectious complications such as anastomotic leakage, pancreatic fistula, or abscesses [13]. Another study highlighted significantly lower rates of pancreatic fistula in RAMIG compared to LG (2.3% vs. 11.4%), which was attributed to the precision of the robotic system, reducing pressure on the pancreas and minimizing parenchymal injury [124].

When CD grade II complications were included, two RCTs by Lu J et al. (7.7% vs. 16.9%, *p* = 0.006) and Ojima T et al. (8.8% vs. 19.7%, *p* = 0.02) demonstrated superior outcomes for RAMIG compared to LG [14,124]. Nonrandomized studies corroborate these findings. Kim HI et al. reported a morbidity rate of 1.1% for both RAMIG and LG, with no significant difference (*p* = 0.999) [98]. A multi-institutional prospective study showed that RAMIG significantly reduced morbidity rates compared to LG (2.45% vs. 6.4%, *p* = 0.0018) [99]. Single-arm prospective studies by Okabe H et al. and Tokunaga M et al. also reported low morbidity rates for RAMIG, with CD grade ≥ IIIa rates of 2.6% and 3.3%, respectively [100,101]. Multi-institutional retrospective studies have similarly reported low morbidity rates for RAMIG (1.3% to 5.4%), which are comparable to those of LG (2.9% to 4.7%) [12,102,130].

Among single-center retrospective studies employing propensity score-matched (PSM) analyses, several have demonstrated the clear advantage of RAMIG. Wang WJ et al. observed morbidity rates of 8.9% for RAMIG versus 17.5% for LG (*p* = 0.002), Shibasaki S. et al. reported 3.7% for RAMIG versus 7.6% for LG (*p* = 0.033), and Omori M et al. noted rates of 1.0% for RAMIG compared to 4.8% for LG (*p* = 0.007) [126,131,132]. Furthermore, Hikage M et al. found that RAMIG significantly reduced CD grade ≥ II intra-abdominal infectious complications compared to LG (4.4% vs. 9.4%, *p* = 0.015), although no significant difference was observed for total complications (RAMIG vs. LG: 13.2% vs. 18.4%, *p* = 0.074) [132].

A meta-analysis conducted by Guerrini G et al. further substantiated these findings, demonstrating significantly lower rates of CD grade ≤ IIIa surgical complications in RAMIG compared to LG. The pooled analysis revealed complication rates of 4.13% (150/3631) for RAMIG versus 6.44% (498/7727) for LG, with an odds ratio (OR) of 0.66 (95% CI 0.49–0.88, *p* = 0.005) [120].

#### 3.4.5. Mortality

No mortality was reported in four RCTs and four prospective studies evaluating RAMIG [13,97,98,99,100,101,121]. Similarly, large-scale, multi-institutional retrospective analyses conducted in East Asia demonstrated exceptionally low mortality rates for RAMIG, ranging from 0% to 0.2%, with no statistically significant differences when compared to LG [12,102,130]. Single-center retrospective studies further supported these findings, with reported mortality rates ranging from 0% to 0.9% for RAMIG, again comparable to those of LG [103,105,106,107,122,126].

However, some studies reported conflicting trends. A multi-institutional retrospective analysis conducted in the United States observed slightly higher mortality rates for both RAMIG (4.5%) and LG (2.7%), though the difference was not statistically significant [133]. Similarly, six meta-analyses indicated a higher mortality rate with RAMIG compared to LG, while Hu LD et al. reported a lower mortality rate for RAMIG, but none of these findings achieved statistical significance [108,109,111,112,114,117,134].

A recent meta-analysis by Guerrini G et al. further substantiated these observations, showing no significant difference in mortality rates between RAMIG and LG. The pooled mortality rates were 0.36% (16/4378) for RAMIG and 0.30% (31/10354) for LG, with an odds ratio (OR) of 1.43 (95% confidence interval [CI]: 0.77–2.65, *p* = 0.25) [120]. The peri-operative outcomes are detailed in Table 2.

#### 3.4.6. Economic Evaluation

The economic evaluation of RAMIG in comparison to LG represents a pivotal aspect in determining its feasibility for widespread adoption. However, a clear cost-effective analysis of robotic surgery for gastric cancer remains challenging due to limited data, with most studies providing only brief mentions of cost. Nonetheless, an extensive literature review was conducted, identifying publications that report substantial findings on the financial aspects of robotic surgery in gastric cancer [12,14,98,106,107]. RAMIG is consistently associated with higher direct costs, primarily driven by the acquisition and maintenance of robotic platforms, such as the da Vinci Surgical System, alongside consumable expenses, including robotic instruments and drapes [136]. In contrast, laparoscopic systems are more cost-efficient, as they are widely available, and their costs have largely been amortized over extended use in most surgical centers. These factors contribute significantly to the procedural cost disparity between the two approaches. However, indirect costs and potential savings associated with RAMIG warrant careful consideration. Robotic systems may contribute to reduced postoperative morbidity, shorter hospital stays, and fewer readmissions, thereby offsetting some of the elevated upfront expenses. Enhanced precision facilitated by robotic platforms, particularly in complex procedures such as lymphadenectomy and esophagojejunostomy, may further minimize complications, including anastomotic leaks and infectious sequelae, potentially reducing the overall cost burden of care. Recent data have provided quantitative insights into these cost differences. For instance, a randomized controlled trial reported a 3% higher hospitalization cost for RAMIG compared to LG (USD 15,953.41 ± 3533.91 vs. USD 12,198.26 ± 2761.27, *p* < 0.001) [137]. While this difference reflects the elevated direct costs of robotic surgery, it also highlights the potential for cost convergence as robotic systems become increasingly adopted, competition among manufacturers intensifies, and procedural efficiencies improve. Moreover, the shorter learning curve associated with RAMIG for surgeons with prior laparoscopic experience may contribute to further cost reductions by enhancing operative efficiency and reducing resource utilization during the training phase.

Regional disparities in healthcare systems also play a critical role in the economic assessment of RAMIG. Much of the current cost-related data derives from Asian centers, where billing practices and reimbursement models differ substantially from those in Western healthcare systems. Consequently, the financial viability of RAMIG may vary across regions, influenced by factors such as labor costs, procedural reimbursement policies, and infrastructure availability [12,14,98,106,107]. Despite these challenges, the broader implementation of robotic systems in Europe and North America could facilitate economies of scale, narrowing cost differentials over time.

Future research should prioritize multicenter studies examining both direct and indirect costs alongside extended follow-up data on oncological efficacy, quality of life, and recurrence rates. Such evidence is essential to establish robust, evidence-based guidelines for assessing the economic sustainability of RAMIG and supporting its equitable integration into clinical practice for the surgical management of gastric cancer.

#### 3.4.7. Oncological Outcomes

The number of studies evaluating long-term outcomes in RAMIG has grown alongside reports on short-term outcomes. Nine studies and one meta-analysis were included in the assessment of long-term oncological outcomes [12,104,106,107,120,132,138,139,140,141]. Among these, only one prospective study specifically evaluated long-term outcomes following RAMIG. Hikage et al. reported highly favorable results, with 5-year OS and RFS rates of 96.7%, despite 12.5% of the patient cohort having advanced gastric cancer [132].

Two multi-institutional retrospective studies further analyzed long-term outcomes. Li et al. found that the 3-year and 5-year OS and DFS rates were comparable between RAMIG and LG [12]. In contrast, another study demonstrated significantly better 3-year OS rates for RAMIG compared to LG (96.3% vs. 89.6%, *p* = 0.009) using the inverse probability of treatment weighting method. Although a trend toward improved 3-year RFS was observed for RAMIG (92.3% vs. 87.2%), it did not reach statistical significance (*p* = 0.073) [138]. Subgroup analyses revealed that RAMIG significantly improved 3-year OS (99.7% vs. 94.4%, *p* = 0.004) and 3-year RFS (99.7% vs. 93.7%, *p* = 0.003) rates in patients with pathological stage IA disease [138]. Propensity score matching analysis further confirmed the superior 3-year OS (97.1% vs. 89.2%; *p* < 0.001) and RFS (94.2% vs. 86.7%; *p* = 0.002) rates in the RAMIG group compared to LG [138].

Six single-center retrospective studies also compared long-term oncological outcomes between RAMIG and LG. Most of these studies found no significant differences in 3-year or 5-year OS and RFS rates between the two approaches [104,106,107,139,140,141]. However, one study highlighted significantly improved 5-year OS (70.4% vs. 50.2%, *p* = 0.039) and 5-year RFS (74.1% vs. 44.5%, *p* = 0.005) rates for RAMIG in patients with pStage II/III GC after propensity score matching [141] (Table 3).

A meta-analysis further examined recurrence rates, reporting a lower but not statistically significant recurrence rate in the RAMIG group compared to the LG group (9.9% vs. 13.5%, *p* = 0.25) [120].

#### 3.4.8. Learning Curve

One proposed advantage of RAMIG is its relatively shorter learning curve compared to LG, particularly for surgeons with prior experience in laparoscopic surgery. Evidence suggests that RAMIG can be performed safely during the initial phase when conducted by surgeons already proficient in LG techniques. Retrospective studies and systematic reviews have shown that experienced gastric cancer surgeons typically achieve competency in RAMIG after approximately 11–25 cases [100,106,122,123,124,130,131,142,143], whereas LG requires a longer learning period, with 40–60 cases needed to reach proficiency [5,107,139,144,145].

Zhou et al. reported that two surgeons with prior LG experience reached a learning plateau for RAMIG after 12 and 14 cases, as assessed using the cumulative summation score method [145]. Similarly, Park et al. demonstrated that three experienced laparoscopic surgeons achieved stable operative times after 6, 9.6, and 18.1 cases, respectively, using a nonlinear least-squares analysis [123]. Huang et al. further compared the learning curves for RAMIG and LG, showing that RAMIG operative and docking times stabilized after 25 cases, whereas LG required approximately 41 procedures for operative times to plateau [146].

A multi-institutional retrospective study by Shimoike et al. evaluated surgeons transitioning to RAMIG after achieving certification under the Endoscopic Surgical Skill Qualification System (ESSQS), which validates expertise in LG. Among 20 surgeons, most had performed ≥100 LG procedures; however, at least 11 cases of RAMIG were required to achieve stable operative times and reduce surgeon fatigue. Interestingly, prior LG experience did not significantly impact operative time or morbidity rates in RAMIG [130].

The learning curve for younger generation surgeons, who started RAMIG after acting as assistant surgeons in at least 50 procedures, has also been analyzed. Despite being early in their RAMIG experience, these surgeons—having acquired ESSQS certification—achieved learning plateaus after 5, 7, 7, 8, and 11 cases (median: 7 cases) [143]. This suggests that prior exposure as an assistant and early familiarity with robotic systems significantly shorten the learning curve for RAMIG.

Notably, there is currently no direct evidence evaluating the learning curve for RAMIG among surgeons without prior LG experience. While such an analysis would provide valuable insights, it remains challenging due to the widespread adoption of LG as the standard minimally invasive approach in recent years.

Collectively, these findings underscore the shorter and more manageable learning curve of RAMIG compared to LG, especially for surgeons with substantial experience in LG or prior exposure to robotic systems. The enhanced visualization, tremor-filtered instrumentation, and ergonomic advantages of robotic platforms may contribute to this improved adaptability, ultimately facilitating quicker skill acquisition.

### 3.5. New Technologies: Image Guided Surgery

Fluorescent-guided surgery has significantly advanced over the past few years, contributing to safer and more precise procedures across various medical specialties. In gastric cancer, this technique shows promise in multiple stages of the operation. The primary applications of fluorescent image-guided surgery include the identification of lymphatic structures for accurate lymphadenectomy and sentinel node biopsy, tumor localization, perigastric vessel visualization, and intraoperative angiography. Currently, the da Vinci Xi^®^ robotic system, developed by Intuitive Surgical Inc. (Sunnyvale, CA, USA), incorporates an integrated fluorescence imaging technology known as Firefly^®^. More recently, CMR Surgical Ltd. (Cambridge, UK) announced the development of vLimeLite™, a new integrated fluorescence system for its Versius^®^ Plus surgical robotic platform.

#### 3.5.1. Near Infrared Fluorescent Guided Lymphadenectomy

It has been established that standard D2 lymphadenectomy should be considered the gold standard for the treatment of locally advanced gastric cancer [147,148]. Intraoperative visualization of lymphatic vessels can facilitate proper lymph node dissection. Near-infrared fluorescence (NIRF)-guided lymphography has been shown to enhance lymph node visualization, increase the number of retrieved lymph nodes during lymphadenectomy, and potentially allow for tailored lymphadenectomy in cases of nonstandard lymph node visualization outside the classic D2 template [149,150,151,152].

In a study by Jeon et al., a comparison of standard laparoscopy, indocyanine green (ICG)-guided laparoscopy, and ICG-guided robotic gastrectomy demonstrated that the robotic ICG-guided approach achieved the highest rate of proper lymphadenectomy [153]. Notably, in obese patients, where lymphadenectomy is typically more challenging, ICG-guided laparoscopic and robotic gastrectomy resulted in the resection of a greater number of lymph nodes. These approaches also demonstrated a significantly higher rate of retrieval of 16 or more lymph nodes, as well as 30 or more lymph nodes, compared to non-ICG-guided techniques [154].

A meta-analysis of NIRF-guided lymphadenectomy in robotic gastric cancer resection, based on five studies and 312 patients, found that the fluorescent-guided group retrieved a significantly higher number of lymph nodes. Moreover, this group experienced a shorter operative time [155]. However, in the Danish trial examining NIRF lymphography for gastroesophageal junction cancers, while more lymph nodes were retrieved in the fluorescence group, none of the additional lymph nodes were metastatic [156].

The recent phase 3 randomized clinical trial by Chen et al. demonstrated that the mean number of lymph nodes retrieved was significantly greater in the ICG group (50.5 vs. 42.0). Furthermore, both OS and DFS were significantly improved in the ICG group. Interestingly, the overall recurrence rate was considerably lower in the ICG group (18.8% vs. 31%) [157].

#### 3.5.2. Near-Infrared Fluorescence-Guided Sentinel Node Biopsy

D2 lymphadenectomy remains the gold standard for the treatment of advanced gastric cancer; however, less extensive resections may be appropriate in early-stage disease. Due to the complex lymphatic drainage of the stomach, the concept of sentinel nodes is still under investigation. Several studies have described this approach in both open and laparoscopic surgeries [158]. In a preclinical animal model using the Da Vinci Si system, fluorescent sentinel node biopsy with ICG and mannose-labeled magnetic nanoparticles was successfully tested [159]. Additionally, sentinel node biopsy has been employed to preserve pyloric lymph nodes during robotic proximal gastrectomy, as demonstrated by Ikoma et al. [160].

#### 3.5.3. Tumor Localization

The injection site for lymphography has been utilized as a landmark for tumor localization in early-stage gastric cancer to ensure proper margins during partial gastrectomy. Liu et al. used this approach to obtain adequate resection margins [135]. For instance, Nakanishi et al. described a method in which 0.1 mL of ICG was endoscopically injected 1 cm proximal to the tumor during preoperative preparation [161]. During robotic gastrectomy with the Firefly^®^ mode, the fluorescent signal from the injection site was used to guide resections, with surgeons aiming to maintain a minimum of a 2 cm margin by resecting at the edge of the fluorescent signal.

#### 3.5.4. Perigastric Vessel Localization

The localization of perigastric vessels plays a crucial role in ensuring vascular preservation during gastrectomy. In a study by Kim et al., ICG was injected immediately after right gastroepiploic vein ligation during laparoscopic and robotic gastrectomies to visualize the infrapyloric artery, crucial for pylorus-preserving gastrectomy, as well as the accessory splenic artery, necessary to prevent inferior polar infarction of the spleen [162]. The infrapyloric artery was visualized in 80% of cases, while the accessory splenic artery was detected in all cases. Lee et al. proposed an approach to localize the accessory left hepatic artery in 31 patients undergoing laparoscopic or robotic surgery. After clamping the artery near the left hepatic lobe, ICG was injected intravenously, and reduced fluorescence in the left hepatic lobe was observed to confirm its location [163].

Robotic distal gastrectomy has also been employed to mitigate the risk of remnant gastric ischemia associated with distal gastrectomy and distal pancreatectomy. Ito et al. demonstrated the visualization of the left inferior phrenic artery, which was found to sufficiently perfuse the remnant stomach even after splenic artery ligation [164].

#### 3.5.5. Angiography

Anastomotic leakage remains one of the most serious complications following gastrectomy, with reported rates ranging from 1.2% to 6.7% [158,165,166]. Intraoperative ICG-based fluorescent angiography is a promising method to predict and potentially prevent anastomotic leakage. Hayakawa et al. evaluated blood flow in the duodenal wall using the Firefly^®^ system during distal gastrectomy [167]. Among 55 patients, 10 were found to have insufficient blood supply, necessitating additional resection of the duodenal stump. Postoperative outcomes were comparable between patients with good and insufficient vascularization. Interestingly, patients with inadequate blood supply had a higher prevalence of aberrant branching of the left hepatic artery compared to those with adequate vascularization.

### 3.6. Current Achievements, Remaining Barriers, and Future Perspectives

The evolution of RAMIG marks a pivotal advancement in gastric cancer surgery, combining technological precision with clinical feasibility. The evidence synthesized in this review underscores several notable advantages of RAMIG over LG, including reduced intraoperative blood loss, lower morbidity rates, and a significantly shorter learning curve for surgeons proficient in laparoscopic techniques [13,138]. Enhanced three-dimensional visualization, tremor-filtered instruments, and greater dexterity offered by robotic platforms have enabled safer, more precise lymphadenectomy, particularly in anatomically complex regions, such as the splenic hilum and around the celiac axis. Furthermore, advancements in reconstruction techniques, including robotic hand-sewn esophagojejunostomy, have improved procedural outcomes and minimized anastomotic complications [102,126]. These technical benefits have translated into improved short-term outcomes, with evidence suggesting comparable or superior oncological adequacy in lymph node retrieval and margin status compared to LG [12,106,120].

Nevertheless, several challenges persist. The financial burden of RAMIG, primarily driven by system acquisition, maintenance costs, and consumables, remains a major limitation, particularly in Western healthcare settings where cost effectiveness plays a critical role in surgical decision-making [136,137]. Encouragingly, recent studies show a narrowing cost gap between RAMIG and LG, suggesting that increasing adoption, system familiarity, and competition among robotic platforms may help drive costs down over time. Despite the promising short-term outcomes, long-term oncological efficacy, including OS and RFS, remains to be validated through large-scale, multicenter RCTs with extended follow-up periods [138].

This study has several limitations inherent to its design as a literature review; therefore, relying on the available literature on reported data and the unavoidable issue of bias, which may be a part of the individual studies analyzed. However, meticulous attention to detail in our review and analysis of the literature with strict search and inclusion criteria have minimized these limitations with rigorous methodology.

Looking forward, further research should focus on several critical areas. Firstly, long-term oncological outcomes must be explored across diverse populations, particularly for patients with advanced gastric cancer, to confirm the oncological noninferiority or superiority of RAMIG compared to LG and open gastrectomy. Secondly, the integration of emerging technologies such as NIRF-guided lymphadenectomy, real-time vascular assessment, and artificial intelligence-assisted navigation holds significant promise for improving surgical precision and enhancing patient outcomes [149,153,157] Comparative cost-effectiveness studies across healthcare systems in Asia, Europe, and the Americas will also be pivotal in addressing economic concerns and ensuring equitable access to robotic surgery.

## 4. Conclusions

RAMIG has emerged as a transformative technique in gastric cancer surgery, offering significant advantages over traditional laparoscopic approaches. Its enhanced precision, reproducibility, and ability to perform complex procedures, such as intracorporeal hand-sewn esophagojejunostomy, make it a highly effective tool for addressing challenges in total gastrectomy. Current evidence demonstrates improved surgical and oncologic outcomes, positioning RAMIG as a leading approach in minimally invasive gastric cancer treatment. While further long-term studies are required to validate its impact on survival and cost effectiveness, RAMIG represents a paradigm shift in surgical practice, with the potential to set a new standard for complex gastric procedures.

## Figures and Tables

**Table 1:** Inclusion and exclusion criteria for studies evaluated in the comprehensive review.

Criteria	Inclusion	Exclusion
Study design	Comparative or noncomparative studies	Narrative reviews, protocols, commentaries
Population	Patients undergoing robotic or laparoscopic gastrectomy for gastric cancer	Studies with fewer than 10 patients
Intervention	Robot-assisted minimally invasive gastrectomy (RAMIG)	Nongastrectomy surgical procedures
Outcomes	Perioperative, oncological, and economic outcomes	No reporting of key outcomes
Languages	English	Non-English

**Table 2:** Perioperative outcomes of the studies cited in the text.

Reference (Year/Country)	Study Design	Patients for Analysis (*n*)	≥ Stage II (%)	TG or SG (%)	Morbidity (%)	Operative Time (min)	Estimated Blood Loss (mL)	Length of Stay After Procedure (Days)
Kim et al., 2016, South Korea [98]	Prospective	RAMIG: 185 LG: 185	19 10	16 16	1.1 1.1 (*p* = 0.999)	221 178 (*p* < 0.001)	50 55 (*p* = 0.318)	6 6 (*p* = 0.862)
Tokunaga et al., 2016, Japan [101]	Prospective	RAMIG: 120	1	12	3.3	348.5	19	9
Wang et al., 2016, China [97]	RCT	RAMIG: 151 OG: 145	76 79	37 31	2.6 2.8 (*p* = 0.756)	243 192 (*p* = 0.002)	94 153 (*p* < 0.001)	5.6 6.7 (*p* = 0.021)
Pan et al., 2017, China [121]	RCT	RAMIG: 102 LG: 61	78 89	65 74	1.0 6.6 (ND)	153 152 (*p* = 0.717)	41 84 (*p* < 0.001)	3.8 5.4 (*p* < 0.001)
Okabe et al., 2019, Japan [100]	Prospective	RAMIG: 115	30	37	2.6	372	15	12
Uyama et al., 2019, Japan [99]	Prospective	RAMIG: 326	12	22	2.45	313	20	9
Wang et al., 2019, China [126]	Retrospective	RAMIG: 354 LG: 354	76 76	43 44	8.9 17.5 (*p* = 0.002)	242 238 (*p* = 0.246)	149 144 (*p* = 0.311)	10.2 11.6 (*p* < 0.001)
Ryan et al., 2020, USA [133]	Retrospective	RAMIG: 631 LG: 1262	66 66	28 28	ND	ND	ND	10.2 11.6 (*p* < 0.001)
Shibasaki et al., 2020, Japan [103]	Retrospective	RAMIG: 354 LG: 354	38 37	30 29	3.7 7.6 (*p* = 0.033)	360 347 (*p* = 0.001)	37 28 (*p* = 0.005)	12 13 (*p* = 0.001)
Li et al., 2020, China [135]	Retrospective	RAMIG: 1776 LG: 1776	35 35	31 31	2.5 2.9	248.5 220 (*p* < 0.001)	127 143 (*p* < 0.001)	9.2 9.3 (*p* = 0.371)
Guerrini et al., 2020, Italy [120]	Meta-Analysis	RAMIG: 5402 LG: 12310	ND	ND	ND	267.34 220.48	96.69 112.72	9 9 (*p* < 0.11)
Ojima et al., 2021, Japan [13]	RCT	RAMIG: 113 LG: 117	42 40	41 32	5.3 16.2 (*p* = 0.01)	297 245 (*p* = 0.001)	25 25 (*p* = 0.18)	12 13 (*p* = 0.93)
Lu et al., 2021, China [14]	RCT	RAMIG: 141 LG: 142	ND	0 0	1.4 1.4	201 182 (*p* < 0.001)	41 56 (*p* = 0.045)	7.9 8.2 (*p* = 0.062)
Suda et al., 2022, Japan [102]	Retrospective	RAMIG: 2671 LG: 2671	ND	14 14	4.9 3.9 (*p* = 0.084)	354 268 (*p* < 0.001)	20 15 (*p* = 0.149)	10 11 (*p* < 0.001)
Shimoike et al., 2022, Japan [103]	Retrospective	RAMIG: 336	33	24	5.4	370	0	10

OG: open gastrectomy; RAMIG: robotic gastrectomy; SG: subtotal gastrectomy; TG: total gastrectomy; RCT: randomized-control trial; ND: not declared.

**Table 3:** Summary of oncological outcomes in robotic versus laparoscopic gastrectomy studies.

Author(s)	Study Type	Sample Size	Oncological Outcomes	*p*-Value	Key Findings
Li et al. [12]	Multi-institutional Retrospective Study	3552	Three-year OS and DFS comparable between RAMIG and LG.	Nonsignificant.	RG associated with less blood loss, more retrieved lymph nodes, and fewer complications.
Tian Y et al. [106]	Propensity Score Matching	1686	No statistically significant differences in 3-year OS (81.2% vs. 80.3%) or RFS (76.6% vs. 77.0%) between RG and LG.	OS: 0.648; RFS: 0.951	RG associated with higher lymph node retrieval and fewer blood losses but longer operation times and higher costs. Patients with advanced gastric cancer showed greater benefit from RG.
Gao G et al. [107]	Propensity Score Matching	1164	No significant differences in 3-year OS (75.5% vs. 73.1%) or 3-year DFS (72.9% vs. 71.4%) between RADG and LADG.	OS: 0.471; DFS: 0.763	RADG associated with less blood loss, greater lymph node retrieval, and higher costs. Benefits were more evident in high BMI patients.
Guerrini G.P et al. [120]	Meta-analysis	17,712	Comparable oncological outcomes between RG and LG, including recurrence rate and resection margins. RG showed a higher mean number of retrieved lymph nodes (MD 1.84).	LN retrieval: 0.0003; Recurrence: NS	RG demonstrated better short-term surgical outcomes, including reduced intraoperative blood loss and lower Dindo-Clavien ≥ 3 complications.
Hikage et al. [132]	Prospective Study	120	Five-year OS: 96.7%, RFS: 96.7%, DSS: 99.2%. For distal/pylorus-preserving gastrectomy, 5-year OS, RFS: 98.1%, DSS: 100%.	Non-significant.	Long-term outcomes of RG are comparable to laparoscopic and open gastrectomy when performed by experienced surgeons.
Suda K et al. [138]	Retrospective Study	1127	3yOS: 96.3% (RG) vs. 89.6% (LG) (HR 0.34, *p* = 0.009). No significant difference in 3yRFS (*p* = 0.073). RG improved 3yOS and 3yRFS in pStage IA.	0.009	RG shows improved 3-year OS and RFS in early-stage gastric cancer (Stage I/II), with no difference in recurrence patterns.
Obama K et al. [139]	Propensity Score Matching	837	No significant difference in 5-year OS (93.3% vs. 91.6%) and RFS (90.7% vs. 90.5%) between RG and LG (*p* = 0.4112 and *p* = 0.8733).	0.4112 & 0.8733	No difference in long-term oncological outcomes. Robotic surgery shows similar outcomes to laparoscopic surgery.
Gao Y et al. [140]	Propensity Score Matching	502	No significant difference in 3-year OS (RAG 76.1% vs. LAG 81.7%) and RFS (RAG 73.0% vs. LAG 67.6%) between RAG and LAG (*p* = 0.118 & *p* = 0.297). After propensity score matching, similar results were observed.	0.118 & 0.297	Both RAG and LAG showed similar short-term recovery and long-term oncological outcomes for AGC patients. RAG had longer operating times and higher costs.
Nakauchi M et al. [141]	Retrospective Cohort Study	814	Five-year overall survival (80.3%) and recurrence-free survival (78.2%) for patients who underwent minimally invasive R0 gastrectomy. Robotic gastrectomy was an independent positive predictor for recurrence-free survival in stage II/III (HR: 0.56, *p* = 0.035).	0.035	Age, ASA status, gastrectomy type, and pathological T and N status were significant prognostic factors. The robotic approach improved long-term outcomes, especially for advanced stages.

OS: overall survival; DFS: disease-free survival; RAMIG: robot-assisted minimally invasive gastrectomy; LG: laparoscopic gastrectomy; RG: robotic gastrectomy; RFS: recurrence-free survival; RADG: robot-assisted distal gastrectomy; LADG: laparoscopic-assisted distal gastrectomy; LN: lymph node; DSS: disease-specific survival; HR: hazard ratio; ASA: American Society of Anesthesiologists; R0: complete resection.

## Data Availability

The datasets generated and/or analyzed during this study are not publicly available but may be obtained from the corresponding author upon reasonable request.

## Abbreviations

RAMIG	Robot-assisted minimally invasive gastrectomy
LG	Laparoscopic Gastrectomy
OG	Open Gastrectomy
LDG	Laparoscopic Distal Gastrectomy
LTG	Laparoscopic Total Gastrectomy
LATG	Laparoscopic-Assisted Total Gastrectomy
TLTG	Total Laparoscopic Total Gastrectomy
CS	Circular Stapler
LS	Linear Stapler
HS	Hand-Sewn

## References

[1] Baral S., Arawker M.H., Sun Q., Jiang M., Wang L., Wang Y., Ali M., Wang D. (2022). Robotic Versus Laparoscopic Gastrectomy for Gastric Cancer: A Mega Meta-Analysis. Front. Surg..

[2] Son T., Hyung W.J. (2016). Laparoscopic Gastric Cancer Surgery: Current Evidence and Future Perspectives. World J. Gastroenterol..

[3] Giulianotti P.C., Coratti A., Angelini M., Sbrana F., Cecconi S., Balestracci T., Caravaglios G. (2003). Robotics in General Surgery: Personal Experience in a Large Community Hospital. Arch. Surg..

[4] Hashizume M., Sugimachi K. (2003). Robot-Assisted Gastric Surgery. Surg. Clin. North. Am..

[5] Son T., Hyung W.J. (2015). Robotic Gastrectomy for Gastric Cancer. J. Surg. Oncol..

[6] Marano A., Young Choi Y., Hyung W.J., Min Kim Y., Kim J., Noh S.H. (2013). Robotic versus Laparoscopic versus Open Gastrectomy: A Meta-Analysis. J. Gastric Cancer.

[7] Son T., Hyung W.J., Lee J.H., Kim Y.M., Noh S.H. (2014). Minimally Invasive Surgery for Serosa-Positive Gastric Cancer (PT4a) in Patients with Preoperative Diagnosis of Cancer without Serosal Invasion. Surg. Endosc..

[8] Woo Y., Choi G.H., Min B.S., Hyung W.J. (2014). Novel Application of Simultaneous Multi-Image Display during Complex Robotic Abdominal Procedures. BMC Surg..

[9] Marano L., Fusario D., Savelli V., Marrelli D., Roviello F. (2021). Robotic versus Laparoscopic Gastrectomy for Gastric Cancer: An Umbrella Review of Systematic Reviews and Meta-Analyses. Updates Surg..

[10] Park S.S., Kim C.S., Mok Y.J., Kim S.J., Kim H. (2005). Il Gastric Cancer Confined to the Muscularis Propria: A Possible Candidate for Laparoscopic Surgery or Adjuvant Therapy. Scand. J. Gastroenterol..

[11] Hur H., Hae M.J., Kim W. (2008). Laparoscopy-Assisted Distal Gastrectomy with D2 Lymphadenectomy for T2b Advanced Gastric Cancers: Three Years’ Experience. J. Surg. Oncol..

[12] Li Z.Y., Zhou Y.B., Li T.Y., Li J.P., Zhou Z.W., She J.J., Hu J.K., Qian F., Shi Y., Tian Y.L. (2023). Robotic Gastrectomy Versus Laparoscopic Gastrectomy for Gastric Cancer: A Multicenter Cohort Study of 5402 Patients in China. Ann Surg.

[13] Ojima T., Nakamura M., Hayata K., Kitadani J., Katsuda M., Takeuchi A., Tominaga S., Nakai T., Nakamori M., Ohi M. (2021). Short-Term Outcomes of Robotic Gastrectomy vs. Laparoscopic Gastrectomy for Patients with Gastric Cancer: A Randomized Clinical Trial. JAMA Surg..

[14] Lu J., Zheng C.H., Xu B.B., Xie J.W., Wang J.B., Lin J.X., Chen Q.Y., Cao L.L., Lin M., Tu R.H. (2021). Assessment of Robotic Versus Laparoscopic Distal Gastrectomy for Gastric Cancer: A Randomized Controlled Trial. Ann. Surg..

[15] Alhossaini R.M., Altamran A.A., Seo W.J., Hyung W.J. (2017). Robotic Gastrectomy for Gastric Cancer: Current Evidence. Ann. Gastroenterol. Surg..

[16] Xuan Y., Hur H., Byun C.S., Han S.U., Cho Y.K. (2013). Efficacy of Intraoperative Gastroscopy for Tumor Localization in Totally Laparoscopic Distal Gastrectomy for Cancer in the Middle Third of the Stomach. Surg. Endosc..

[17] Kim H.I., Hyung W.J., Lee C.R., Lim J.S., An J.Y., Cheong J.H., Choi S.H., Noh S.H. (2011). Intraoperative Portable Abdominal Radiograph for Tumor Localization: A Simple and Accurate Method for Laparoscopic Gastrectomy. Surg. Endosc..

[18] Huang K.H., Lan Y.T., Fang W.L., Chen J.H., Lo S.S., Hsieh M.C., Li A.F.Y., Chiou S.H., Wu C.W. (2012). Initial Experience of Robotic Gastrectomy and Comparison with Open and Laparoscopic Gastrectomy for Gastric Cancer. J. Gastrointest. Surg..

[19] Hyung W.J., Lim J.S., Cheong J.H., Kim J., Choi S.H., Song S.Y., Noh S.H. (2005). Intraoperative Tumor Localization Using Laparoscopic Ultrasonography in Laparoscopic-Assisted Gastrectomy. Surg. Endosc. Other Interv. Tech..

[20] De Jongh C., Cianchi F., Kinoshita T., Kingma F., Piccoli M., Dubecz A., Kouwenhoven E., Van Det M., Mala T., Coratti A. (2024). Surgical Techniques and Related Perioperative Outcomes After Robot-Assisted Minimally Invasive Gastrectomy (RAMIG): Results From the Prospective Multicenter International Ugira Gastric Registry. Ann. Surg..

[21] Makuuchi R., Terashima M., Terada M., Mizusawa J., Kita R., Tokunaga M., Omori T., Ojima T., Ehara K., Watanabe M. (2023). Randomized Controlled Phase III Trial to Investigate Superiority of Robot-Assisted Gastrectomy over Laparoscopic Gastrectomy for Clinical Stage T1-4aN0-3 Gastric Cancer Patients (JCOG1907, MONA LISA Study): A Study Protocol. BMC Cancer.

[22] Kitano S., Iso Y., Moriyama M., Sugimachi K. (1994). Laparoscopy-Assisted Billroth I Gastrectomy. Surg. Laparosc. Endosc..

[23] Lee H.-J., Hyung W.J., Yang H.-K., Han S.U., Park Y.-K., An J.Y., Kim W., Kim H.-I., Kim H.-H., Ryu S.W. (2019). Short-Term Outcomes of a Multicenter Randomized Controlled Trial Comparing Laparoscopic Distal Gastrectomy With D2 Lymphadenectomy to Open Distal Gastrectomy for Locally Advanced Gastric Cancer (KLASS-02-RCT). Ann. Surg..

[24] Kim W., Kim H.-H., Han S.-U., Kim M.-C., Hyung W.J., Ryu S.W., Cho G.S., Kim C.Y., Yang H.-K., Park D.J. (2016). Decreased Morbidity of Laparoscopic Distal Gastrectomy Compared with Open Distal Gastrectomy for Stage I Gastric Cancer: Short-Term Outcomes From a Multicenter Randomized Controlled Trial (KLASS-01). Ann. Surg..

[25] Katai H., Mizusawa J., Katayama H., Takagi M., Yoshikawa T., Fukagawa T., Terashima M., Misawa K., Teshima S., Koeda K. (2017). Short-Term Surgical Outcomes from a Phase III Study of Laparoscopy-Assisted versus Open Distal Gastrectomy with Nodal Dissection for Clinical Stage IA/IB Gastric Cancer: Japan Clinical Oncology Group Study JCOG0912. Gastric Cancer.

[26] Shi Y., Xu X., Zhao Y., Qian F., Tang B., Hao Y., Luo H., Chen J., Yu P. (2018). Short-Term Surgical Outcomes of a Randomized Controlled Trial Comparing Laparoscopic versus Open Gastrectomy with D2 Lymph Node Dissection for Advanced Gastric Cancer. Surg. Endosc..

[27] Yu J., Huang C., Sun Y., Su X., Cao H., Hu J., Wang K., Suo J., Tao K., He X. (2019). Effect of Laparoscopic vs. Open Distal Gastrectomy on 3-Year Disease-Free Survival in Patients with Locally Advanced Gastric Cancer: The CLASS-01 Randomized Clinical Trial. JAMA.

[28] Meinero M. (1994). Laparoscopic Surgery—The Nineties.

[29] Ramos M.F.K.P., Pereira M.A., Dias A.R., Ribeiro U.J., Zilberstein B., Nahas S.C. (2021). Laparoscopic Gastrectomy for Early and Advanced Gastric Cancer in a Western Center: A Propensity Score-Matched Analysis. Updates Surg..

[30] Yuu K., Tsuchihashi K., Toyoda S., Kawasaki M., Kameyama M. (2019). Laparoscopic vs. Open Distal Gastrectomy for Advanced Gastric Cancer in Elderly Patients: A Retrospective Study. Mini-Invasive Surg..

[31] Kelly K.J., Selby L., Chou J.F., Dukleska K., Capanu M., Coit D.G., Brennan M.F., Strong V.E. (2015). Laparoscopic Versus Open Gastrectomy for Gastric Adenocarcinoma in the West: A Case-Control Study. Ann. Surg. Oncol..

[32] Popiela T., Kulig J., Kolodziejczyk P., Sierzega M. (2002). Long-Term Results of Surgery for Early Gastric Cancer. Br. J. Surg..

[33] Chevallay M., Jung M., Berlth F., Seung-Hun C., Morel P., Mönig S. (2019). Laparoscopic Surgery for Gastric Cancer: The European Point of View. J. Oncol..

[34] Zhu Z., Li L., Xu J., Ye W., Zeng J., Chen B., Huang Z. (2020). Laparoscopic versus Open Approach in Gastrectomy for Advanced Gastric Cancer: A Systematic Review. World J. Surg. Oncol..

[35] Wei Y., Yu D., Li Y., Fan C., Li G. (2018). Laparoscopic versus Open Gastrectomy for Advanced Gastric Cancer: A Meta-Analysis Based on High-Quality Retrospective Studies and Clinical Randomized Trials. Clin. Res. Hepatol. Gastroenterol..

[36] Azagra J.S., Goergen M., De Simone P., Ibañez-Aguirre J. (1999). Minimally Invasive Surgery for Gastric Cancer. Surg. Endosc..

[37] Umemura A., Koeda K., Sasaki A., Fujiwara H., Kimura Y., Iwaya T., Akiyama Y., Wakabayashi G. (2015). Totally Laparoscopic Total Gastrectomy for Gastric Cancer: Literature Review and Comparison of the Procedure of Esophagojejunostomy. Asian J. Surg..

[38] Azagra J.S., Goergen M., Arru L., Facy O. (2013). Total Gastrectomy for Locally Advanced Cancer: The Pure Laparoscopic Approach. Gastroenterol. Rep. (Oxf.).

[39] Azagra J.S., Sarriugarte A., Ibañez F.J. (2018). Current Status of Gastrectomy for Cancer: “Less Is Often More”. Cir. Esp..

[40] Lee S., Lee H., Song J.H., Choi S., Cho M., Son T., Kim H.-I., Hyung W.J. (2020). Intracorporeal Esophagojejunostomy Using a Linear Stapler in Laparoscopic Total Gastrectomy: Comparison with Circular Stapling Technique. BMC Surg..

[41] Huang C., Zhao J., Liu Z., Huang J., Zhu Z. (2020). Esophageal Suspension Method for Hand-Sewn Esophagojejunostomy After Totally Laparoscopic Total Gastrectomy: A Simple, Safe, and Feasible Suturing Technique. Front. Oncol..

[42] Norero E., Muñoz R., Ceroni M., Manzor M., Crovari F., Gabrielli M. (2017). Two-Layer Hand-Sewn Esophagojejunostomy in Totally Laparoscopic Total Gastrectomy for Gastric Cancer. J. Gastric Cancer.

[43] Chen K., Wu D., Pan Y., Cai J.-Q., Yan J.-F., Chen D.-W., Maher H., Mou Y.-P. (2016). Totally Laparoscopic Gastrectomy Using Intracorporeally Stapler or Hand-Sewn Anastomosis for Gastric Cancer: A Single-Center Experience of 478 Consecutive Cases and Outcomes. World J. Surg. Oncol..

[44] So K.O., Park J.-M. (2011). Totally Laparoscopic Total Gastrectomy Using Intracorporeally Hand-Sewn Esophagojejunostomy. J. Gastric Cancer.

[45] Voeten D.M., Busweiler L.A.D., van der Werf L.R., Wijnhoven B.P.L., Verhoeven R.H.A., van Sandick J.W., van Hillegersberg R., van Berge Henegouwen M.I. (2021). Outcomes of Esophagogastric Cancer Surgery During Eight Years of Surgical Auditing by the Dutch Upper Gastrointestinal Cancer Audit (DUCA). Ann. Surg..

[46] Trapani R., Rausei S., Reddavid R., Degiuli M. (2020). Risk Factors for Esophago-Jejunal Anastomosis Leakage after Total Gastrectomy for Cancer. A Multicenter Retrospective Study of the Italian Research Group for Gastric Cancer. Eur. J. Surg. Oncol..

[47] Ebihara Y., Kurashima Y., Tanaka K., Nakanishi Y., Asano T., Noji T., Nakamura T., Murakami S., Tsuchikawa T., Okamura K. (2021). A Multicenter Retrospective Study Comparing Surgical Outcomes Between the Overlap Method and Functional Method for Esophagojejunostomy in Laparoscopic Total Gastrectomy: Analysis Using Propensity Score Matching. Surg. Laparosc. Endosc. Percutan Tech..

[48] Inokuchi M., Otsuki S., Fujimori Y., Sato Y., Nakagawa M., Kojima K. (2015). Systematic Review of Anastomotic Complications of Esophagojejunostomy after Laparoscopic Total Gastrectomy. World J. Gastroenterol..

[49] Jeong O., Park Y.K. (2009). Intracorporeal Circular Stapling Esophagojejunostomy Using the Transorally Inserted Anvil (OrVil) after Laparoscopic Total Gastrectomy. Surg. Endosc..

[50] Kim H.-I., Cho I., Jang D.-S., Hyung W.J. (2013). Intracorporeal Esophagojejunostomy Using a Circular Stapler with a New Purse-String Suture Technique during Laparoscopic Total Gastrectomy. J. Am. Coll. Surg..

[51] Kwon I.G., Son Y.-G., Ryu S.W. (2016). Novel Intracorporeal Esophagojejunostomy Using Linear Staplers During Laparoscopic Total Gastrectomy: π-Shaped Esophagojejunostomy, 3-in-1 Technique. J. Am. Coll. Surg..

[52] Du J., Xue H., Zhao L., Hua J., Hu J., Zhang Z. (2019). Intracorporeal Circular-Stapled Anastomosis after Totally Laparoscopic Gastrectomy: A Novel, Simplest u-Shaped Parallel Purse-String Suture Technique. J. Surg. Oncol..

[53] Dulucq J.-L., Wintringer P., Perissat J., Mahajna A. (2005). Completely Laparoscopic Total and Partial Gastrectomy for Benign and Malignant Diseases: A Single Institute’s Prospective Analysis. J. Am. Coll. Surg..

[54] Usui S., Nagai K., Hiranuma S., Takiguchi N., Matsumoto A., Sanada K. (2008). Laparoscopy-Assisted Esophagoenteral Anastomosis Using Endoscopic Purse-String Suture Instrument “Endo-PSI (II)” and Circular Stapler. Gastric Cancer.

[55] Kawaguchi Y., Shiraishi K., Akaike H., Ichikawa D. (2019). Current Status of Laparoscopic Total Gastrectomy. Ann. Gastroenterol. Surg..

[56] Facy O., Arru L., Azagra J.S. (2012). Intestinal Anastomosis after Laparoscopic Total Gastrectomy. J. Visc. Surg..

[57] Xu X., Huang C., Mou Y., Zhang R., Pan Y., Chen K., Lu C. (2018). Intra-Corporeal Hand-Sewn Esophagojejunostomy Is a Safe and Feasible Procedure for Totally Laparoscopic Total Gastrectomy: Short-Term Outcomes in 100 Consecutive Patients. Surg. Endosc..

[58] Azagra J.S., Pascotto B., Arru L., Ibañez F.J., Makkai-Popa S.T., Goergen M., Asunción Acosta M., Cuesta M.A., Bruna M. (2021). Hand-Sewn Anastomosis After 95% Gastrectomy, Total Gastrectomy, and Total Gastrectomy Extended to the Distal Esophagus for Gastric Cancer. Atlas of Minimally Invasive Techniques in Upper Gastrointestinal Surgery.

[59] Pascotto B., González González L., Di Saverio S., Arru L., Goergen M., Azagra J.S. (2023). Minimally Invasive Hand-Sewn Barbed Anastomosis After Total and Near-Total Gastrectomy: Standardized Azagra’s Technique. J. Gastrointest. Surg..

[60] Wang Z., Wei Y., Liu X., Li Z., Zhu G., Li Y., Wang K. (2021). Application Value of Hand-Sewn Anastomosis in Totally Laparoscopic Total Gastrectomy for Gastric Cancer. World J. Surg. Oncol..

[61] Sierzega M., Kolodziejczyk P., Kulig J. (2010). Impact of Anastomotic Leakage on Long-Term Survival after Total Gastrectomy for Carcinoma of the Stomach. Br. J. Surg..

[62] Yoo H.M., Lee H.H., Shim J.H., Jeon H.M., Park C.H., Song K.Y. (2011). Negative Impact of Leakage on Survival of Patients Undergoing Curative Resection for Advanced Gastric Cancer. J. Surg. Oncol..

[63] Deguchi Y., Fukagawa T., Morita S., Ohashi M., Saka M., Katai H. (2012). Identification of Risk Factors for Esophagojejunal Anastomotic Leakage after Gastric Surgery. World J. Surg..

[64] Makuuchi R., Irino T., Tanizawa Y., Bando E., Kawamura T., Terashima M. (2019). Esophagojejunal Anastomotic Leakage Following Gastrectomy for Gastric Cancer. Surg. Today.

[65] Schietroma M., Cecilia E.M., Carlei F., Sista F., De Santis G., Piccione F., Amicucci G. (2013). Prevention of Anastomotic Leakage after Total Gastrectomy with Perioperative Supplemental Oxygen Administration: A Prospective Randomized, Double-Blind, Controlled, Single-Center Trial. Ann. Surg. Oncol..

[66] Hyung W.J., Yang H.-K., Han S.-U., Lee Y.-J., Park J.-M., Kim J.J., Kwon O.K., Kong S.H., Kim H.-I., Lee H.-J. (2019). A Feasibility Study of Laparoscopic Total Gastrectomy for Clinical Stage I Gastric Cancer: A Prospective Multi-Center Phase II Clinical Trial, KLASS 03. Gastric Cancer.

[67] Watanabe M., Miyata H., Gotoh M., Baba H., Kimura W., Tomita N., Nakagoe T., Shimada M., Kitagawa Y., Sugihara K. (2014). Total Gastrectomy Risk Model: Data from 20,011 Japanese Patients in a Nationwide Internet-Based Database. Ann. Surg..

[68] Bruce J., Krukowski Z.H., Al-Khairy G., Russell E.M., Park K.G. (2001). Systematic Review of the Definition and Measurement of Anastomotic Leak after Gastrointestinal Surgery. Br. J. Surg..

[69] Budisin N., Budisin E., Golubovic A. (2001). Early Complications Following Total Gastrectomy for Gastric Cancer. J. Surg. Oncol..

[70] Robb W.B., Messager M., Goere D., Pichot-Delahaye V., Lefevre J.H., Louis D., Guiramand J., Kraft K., Mariette C. (2013). Predictive Factors of Postoperative Mortality after Junctional and Gastric Adenocarcinoma Resection. JAMA Surg..

[71] Liu K., Yang K., Zhang W., Chen X., Chen X., Zhang B., Chen Z., Chen J., Zhao Y., Zhou Z. (2016). Changes of Esophagogastric Junctional Adenocarcinoma and Gastroesophageal Reflux Disease Among Surgical Patients During 1988-2012: A Single-Institution, High-Volume Experience in China. Ann Surg.

[72] Kauppila J.H., Lagergren J. (2016). The Surgical Management of Esophago-Gastric Junctional Cancer. Surg. Oncol..

[73] Liakakos T. (2011). Totally Laparoscopic Total Gastrectomy and the Challenge of Esophagojejunostomy. Surg. Endosc..

[74] Kinoshita T., Oshiro T., Ito K., Shibasaki H., Okazumi S., Katoh R. (2010). Intracorporeal Circular-Stapled Esophagojejunostomy Using Hand-Sewn Purse-String Suture after Laparoscopic Total Gastrectomy. Surg. Endosc..

[75] Matsuda T., Iwasaki T., Mitsutsuji M., Hirata K., Maekawa Y., Tsugawa D., Sugita Y., Shimada E., Kakeji Y. (2015). Surgical Outcomes of Intracorporeal Circular-Stapled Esophagojejunostomy Using Modified over-and-over Suture Technique in Laparoscopic Total Gastrectomy. Surg. Endosc..

[76] Omori T., Oyama T., Mizutani S., Tori M., Nakajima K., Akamatsu H., Nakahara M., Nishida T. (2009). A Simple and Safe Technique for Esophagojejunostomy Using the Hemidouble Stapling Technique in Laparoscopy-Assisted Total Gastrectomy. Am. J. Surg..

[77] Hiki N., Fukunaga T., Yamaguchi T., Nunobe S., Tokunaga M., Ohyama S., Seto Y., Muto T. (2007). Laparoscopic Esophagogastric Circular Stapled Anastomosis: A Modified Technique to Protect the Esophagus. Gastric Cancer.

[78] Sano A., Ojima H., Ogawa A., Ogata K., Saito K., Fukasawa T., Sohda M., Fukai Y., Mochida Y., Fukuchi M. (2020). Four Stay-Sutures Method: A Simplified Hand-Sewn Purse-String Suture in Laparoscopic Circular-Stapled Esophagojejunostomy. Surg. Today.

[79] Muneoka Y., Ohashi M., Makuuchi R., Ida S., Kumagai K., Sano T., Nunobe S. (2021). Advantageous Short-Term Outcomes of Esophagojejunostomy Using a Linear Stapler Following Open Total Gastrectomy Compared with a Circular Stapler. World J. Surg..

[80] Chen K., He Y., Cai J.-Q., Pan Y., Wu D., Chen D.-W., Yan J.-F., Maher H., Mou Y.-P. (2016). Comparing the Short-Term Outcomes of Intracorporeal Esophagojejunostomy with Extracorporeal Esophagojejunostomy after Laparoscopic Total Gastrectomy for Gastric Cancer. BMC Surg..

[81] Sun Z., Zheng X., Chen G., Wang L., Sang Q., Xu G., Zhang N. (2020). Aminbuhe Technical Details of and Prognosis for the “China Stitch”, a Novel Technique for Totally Laparoscopic Hand-Sewn Esophagojejunostomy. Biosci. Trends.

[82] Yan J.-F., Chen K., Pan Y., Maher H., Zhu H.-P., Lou S.-M., Wang Y. (2020). Laparoscopic Gastrectomy Using Intracorporeally Hand-Sewn Anastomosis of Esophagojejunostomy, Gastroduodenostomy, or Gastrojejunostomy for Gastric Cancer. Medicine.

[83] Salvador-Rosés H., Escartín A., Muriel P., Santamaría M., González M., Jara J., Vela F., Olsina J.-J. (2023). Robotic versus Open Approach in Total Gastrectomy for Gastric Cancer: A Comparative Single-Center Study of Perioperative Outcomes. J. Robot. Surg..

[84] Hur H., Kim J.Y., Cho Y.K., Han S.-U. (2010). Technical Feasibility of Robot-Sewn Anastomosis in Robotic Surgery for Gastric Cancer. J. Laparoendosc. Adv. Surg. Tech. A.

[85] Parisi A., Ricci F., Trastulli S., Cirocchi R., Gemini A., Grassi V., Corsi A., Renzi C., De Santis F., Petrina A. (2015). Robotic Total Gastrectomy with Intracorporeal Robot-Sewn Anastomosis: A Novel Approach Adopting the Double-Loop Reconstruction Method. Medicine.

[86] De Blasi V., Facy O., Goergen M., Poulain V., De Magistris L., Azagra J.S. (2013). Barbed versus Usual Suture for Closure of the Gastrojejunal Anastomosis in Laparoscopic Gastric Bypass: A Comparative Trial. Obes. Surg..

[87] Facy O., De Blasi V., Goergen M., Arru L., De Magistris L., Azagra J.-S. (2013). Laparoscopic Gastrointestinal Anastomoses Using Knotless Barbed Sutures Are Safe and Reproducible: A Single-Center Experience with 201 Patients. Surg. Endosc..

[88] Morelli L., Furbetta N., Gianardi D., Guadagni S., Di Franco G., Bianchini M., Palmeri M., Masoni C., Di Candio G., Cuschieri A. (2021). Use of Barbed Suture without Fashioning the “Classical” Wirsung-Jejunostomy in a Modified End-to-Side Robotic Pancreatojejunostomy. Surg. Endosc..

[89] Arena A., Degli Esposti E., Cristani G., Orsini B., Moro E., Raimondo D., Del Forno S., Lenzi J., Casadio P., Seracchioli R. (2021). Comparison of Fertility Outcomes after Laparoscopic Myomectomy for Barbed versus Nonbarbed Sutures. Fertil. Steril..

[90] Einarsson J.I., Chavan N.R., Suzuki Y., Jonsdottir G., Vellinga T.T., Greenberg J.A. (2011). Use of Bidirectional Barbed Suture in Laparoscopic Myomectomy: Evaluation of Perioperative Outcomes, Safety, and Efficacy. J. Minim. Invasive Gynecol..

[91] Siedhoff M.T., Yunker A.C., Steege J.F. (2011). Decreased Incidence of Vaginal Cuff Dehiscence after Laparoscopic Closure with Bidirectional Barbed Suture. J. Minim. Invasive Gynecol..

[92] Tsukada T., Kaji M., Kinoshita J., Shimizu K. (2016). Use of Barbed Sutures in Laparoscopic Gastrointestinal Single-Layer Sutures. JSLS.

[93] Peleg D., Ahmad R.S., Warsof S.L., Marcus-Braun N., Sciaky-Tamir Y., Ben Shachar I. (2018). A Randomized Clinical Trial of Knotless Barbed Suture vs. Conventional Suture for Closure of the Uterine Incision at Cesarean Delivery. Am. J. Obs. Gynecol..

[94] Chen K., Pan Y., Cai J.-Q., Xu X.-W., Wu D., Yan J.-F., Chen R.-G., He Y., Mou Y.-P. (2016). Intracorporeal Esophagojejunostomy after Totally Laparoscopic Total Gastrectomy: A Single-Center 7-Year Experience. World J. Gastroenterol..

[95] Woo Y., Hyung W.J., Pak K.H., Inaba K., Obama K., Choi S.H., Noh S.H. (2011). Robotic Gastrectomy as an Oncologically Sound Alternative to Laparoscopic Resections for the Treatment of Early-Stage Gastric Cancers. Arch. Surg..

[96] Pugliese R., Maggioni D., Sansonna F., Costanzi A., Ferrari G.C., Di Lernia S., Magistro C., De Martini P., Pugliese F. (2010). Subtotal Gastrectomy with D2 Dissection by Minimally Invasive Surgery for Distal Adenocarcinoma of the Stomach: Results and 5-Year Survival. Surg. Endosc..

[97] Wang G., Jiang Z., Zhao J., Liu J., Zhang S., Zhao K., Feng X., Li J. (2016). Assessing the Safety and Efficacy of Full Robotic Gastrectomy with Intracorporeal Robot-Sewn Anastomosis for Gastric Cancer: A Randomized Clinical Trial. J. Surg. Oncol..

[98] Kim H.I., Han S.U., Yang H.K., Kim Y.W., Lee H.J., Ryu K.W., Park J.M., An J.Y., Kim M.C., Park S. (2016). Multicenter Prospective Comparative Study of Robotic versus Laparoscopic Gastrectomy for Gastric Adenocarcinoma. Ann. Surg..

[99] Uyama I., Suda K., Nakauchi M., Kinoshita T., Noshiro H., Takiguchi S., Ehara K., Obama K., Kuwabara S., Okabe H. (2019). Clinical Advantages of Robotic Gastrectomy for Clinical Stage I/II Gastric Cancer: A Multi-Institutional Prospective Single-Arm Study. Gastric Cancer.

[100] Okabe H., Obama K., Tsunoda S., Matsuo K., Tanaka E., Hisamori S., Sakai Y. (2019). Feasibility of Robotic Radical Gastrectomy Using a Monopolar Device for Gastric Cancer. Surg. Today.

[101] Tokunaga M., Makuuchi R., Miki Y., Tanizawa Y., Bando E., Kawamura T., Terashima M. (2016). Late Phase II Study of Robot-Assisted Gastrectomy with Nodal Dissection for Clinical Stage I Gastric Cancer. Surg. Endosc..

[102] Suda K., Yamamoto H., Nishigori T., Obama K., Yoda Y., Hikage M., Shibasaki S., Tanaka T., Kakeji Y., Inomata M. (2022). Safe Implementation of Robotic Gastrectomy for Gastric Cancer under the Requirements for Universal Health Insurance Coverage: A Retrospective Cohort Study Using a Nationwide Registry Database in Japan. Gastric Cancer.

[103] Shibasaki S., Suda K., Nakauchi M., Nakamura K., Kikuchi K., Inaba K., Uyama I. (2020). Non-Robotic Minimally Invasive Gastrectomy as an Independent Risk Factor for Postoperative Intra-Abdominal Infectious Complications: A Single-Center, Retrospective and Propensity Score-Matched Analysis. World J. Gastroenterol..

[104] Hikage M., Fujiya K., Kamiya S., Tanizawa Y., Bando E., Notsu A., Mori K., Terashima M. (2021). Robotic Gastrectomy Compared with Laparoscopic Gastrectomy for Clinical Stage I/II Gastric Cancer Patients: A Propensity Score-Matched Analysis. World J. Surg..

[105] Zheng-yan L., Yong-liang Z., Feng Q., Yan S., Pei-wu Y. (2021). Morbidity and Short-Term Surgical Outcomes of Robotic versus Laparoscopic Distal Gastrectomy for Gastric Cancer: A Large Cohort Study. Surg. Endosc..

[106] Tian Y., Cao S., Kong Y., Shen S., Niu Z., Zhang J., Chen D., Jiang H., Lv L., Liu X. (2022). Short- and Long-Term Comparison of Robotic and Laparoscopic Gastrectomy for Gastric Cancer by the Same Surgical Team: A Propensity Score Matching Analysis. Surg. Endosc..

[107] Gao G., Liao H., Jiang Q., Liu D., Li T. (2022). Surgical and Oncological Outcomes of Robotic- versus Laparoscopic-Assisted Distal Gastrectomy with D2 Lymphadenectomy for Advanced Gastric Cancer: A Propensity Score-Matched Analysis of 1164 Patients. World J. Surg. Oncol..

[108] Xiong B., Ma L., Zhang C. (2012). Robotic versus Laparoscopic Gastrectomy for Gastric Cancer: A Meta-Analysis of Short Outcomes. Surg. Oncol..

[109] Chen K., Pan Y., Zhang B., Maher H., Wang X.F., Cai X.J. (2017). Robotic versus Laparoscopic Gastrectomy for Gastric Cancer: A Systematic Review and Updated Meta-Analysis. BMC Surg..

[110] Wang Y., Zhao X., Song Y., Cai A., Xi H., Chen L. (2017). A Systematic Review and Meta-Analysis of Robot-Assisted versus Laparoscopically Assisted Gastrectomy for Gastric Cancer. Medecine.

[111] Bobo Z., Xin W., Jiang L., Quan W., Liang B., Xiangbing D., Ziqiang W. (2019). Robotic Gastrectomy versus Laparoscopic Gastrectomy for Gastric Cancer: Meta-Analysis and Trial Sequential Analysis of Prospective Observational Studies. Surg. Endosc..

[112] Xiong J., Nunes Q.M., Tan C., Ke N., Chen Y., Hu W., Liu X., Mai G. (2013). Comparison of Short-Term Clinical Outcomes between Robotic and Laparoscopic Gastrectomy for Gastric Cancer: A Meta-Analysis of 2495 Patients. J. Laparoendosc. Adv. Surg. Tech..

[113] Hyun M.H., Lee C.H., Kim H.J., Tong Y., Park S.S. (2013). Systematic Review and Meta-Analysis of Robotic Surgery Compared with Conventional Laparoscopic and Open Resections for Gastric Carcinoma. Br. J. Surg..

[114] Zong L., Seto Y., Aikou S., Takahashi T. (2014). Efficacy Evaluation of Subtotal and Total Gastrectomies in Robotic Surgery for Gastric Cancer Compared with That in Open and Laparoscopic Resections: A Meta-Analysis. PLoS ONE.

[115] Chuan L., Yan S., Pei-Wu Y. (2015). Meta-Analysis of the Short-Term Outcomes of Robotic-Assisted Compared to Laparoscopic Gastrectomy. Minim. Invasive Ther. Allied Technol..

[116] Wang Z., Wang Y., Liu Y. (2017). Comparison of Short Outcomes between Laparoscopic and Experienced Robotic Gastrectomy: A Meta-Analysis and Systematic Review. J. Minim. Access Surg..

[117] Hu L., Li X., Wang X., Guo T. (2016). Robotic versus Laparoscopic Gastrectomy for Gastric Carcinoma: A Meta-Analysis of Efficacy and Safety. Asian Pac. J. Cancer Prev..

[118] Kang B.H., Xuan Y., Hur H., Ahn C.W., Cho Y.K., Han S.U. (2012). Comparison of Surgical Outcomes between Robotic and Laparoscopic Gastrectomy for Gastric Cancer: The Learning Curve of Robotic Surgery. J. Gastric Cancer.

[119] Liu H., Kinoshita T., Tonouchi A., Kaito A., Tokunaga M. (2019). What Are the Reasons for a Longer Operation Time in Robotic Gastrectomy than in Laparoscopic Gastrectomy for Stomach Cancer?. Surg. Endosc..

[120] Guerrini G.P., Esposito G., Magistri P., Serra V., Guidetti C., Olivieri T., Catellani B., Assirati G., Ballarin R., Di Sandro S. (2020). Robotic versus Laparoscopic Gastrectomy for Gastric Cancer: The Largest Meta-Analysis. Int. J. Surg..

[121] Pan H.F., Wang G., Liu J., Liu X.X., Zhao K., Tang X.F., Jiang Z.W. (2017). Robotic versus Laparoscopic Gastrectomy for Locally Advanced Gastric Cancer. Surg. Laparosc. Endosc. Percutan Tech..

[122] Omori T., Yamamoto K., Hara H., Shinno N., Yamamoto M., Fujita K., Kanemura T., Takeoka T., Akita H., Wada H. (2022). Comparison of Robotic Gastrectomy and Laparoscopic Gastrectomy for Gastric Cancer: A Propensity Score-Matched Analysis. Surg. Endosc..

[123] Park S.S., Kim M.C., Park M.S., Hyung W.J. (2012). Rapid Adaptation of Robotic Gastrectomy for Gastric Cancer by Experienced Laparoscopic Surgeons. Surg. Endosc..

[124] Suda K., Man-i M., Ishida Y., Kawamura Y., Satoh S., Uyama I. (2015). Potential Advantages of Robotic Radical Gastrectomy for Gastric Adenocarcinoma in Comparison with Conventional Laparoscopic Approach: A Single Institutional Retrospective Comparative Cohort Study. Surg. Endosc..

[125] Tsai S.H., Liu C.A., Huang K.H., Lan Y.T., Chen M.H., Chao Y., Lo S.S., Li A.F.Y., Wu C.W., Chiou S.H. (2017). Advances in Laparoscopic and Robotic Gastrectomy for Gastric Cancer. Pathol. Oncol. Res..

[126] Wang W.J., Li H.T., Yu J.P., Su L., Guo C.A., Chen P., Yan L., Li K., Ma Y.W., Wang L. (2019). Severity and Incidence of Complications Assessed by the Clavien-Dindo Classification Following Robotic and Laparoscopic Gastrectomy for Advanced Gastric Cancer: A Retrospective and Propensity Score-Matched Study. Surg. Endosc..

[127] Noshiro H., Ikeda O., Urata M. (2014). Robotically-Enhanced Surgical Anatomy Enables Surgeons to Perform Distal Gastrectomy for Gastric Cancer Using Electric Cautery Devices Alone. Surg. Endosc..

[128] Dindo D., Demartines N., Clavien P.A. (2004). Classification of Surgical Complications: A New Proposal with Evaluation in a Cohort of 6336 Patients and Results of a Survey. Ann. Surg..

[129] Clavien P.A., Barkun J., De Oliveira M.L., Vauthey J.N., Dindo D., Schulick R.D., De Santibañes E., Pekolj J., Slankamenac K., Bassi C. (2009). The Clavien-Dindo Classification of Surgical Complications: Five-Year Experience. Ann. Surg..

[130] Shimoike N., Nishigori T., Yamashita Y., Kondo M., Manaka D., Kadokawa Y., Itami A., Kanaya S., Hosogi H., Satoh S. (2022). Safety Assessment of Robotic Gastrectomy and Analysis of Surgical Learning Process: A Multicenter Cohort Study. Gastric Cancer.

[131] Shibasaki S., Suda K., Obama K., Yoshida M., Uyama I. (2020). Should Robotic Gastrectomy Become a Standard Surgical Treatment Option for Gastric Cancer?. Surg. Today.

[132] Hikage M., Tokunaga M., Furukawa K., Fujiya K., Kamiya S., Tanizawa Y., Bando E., Terashima M. (2020). Long-Term Outcomes of Robotic Gastrectomy for Clinical Stage I Gastric Cancer: A Single-Center Prospective Phase II Study. Surg. Endosc..

[133] Ryan S., Tameron A., Murphy A., Hussain L., Dunki-Jacobs E., Lee D.Y. (2020). Robotic versus Laparoscopic Gastrectomy for Gastric Adenocarcinoma: Propensity-Matched Analysis. Surg. Innov..

[134] Liao G.X., Xie G.Z., Li R., Zhao Z.H., Sun Q.Q., Du S.S., Ren C., Li G.X., Deng H.J., Yuan Y.W. (2013). Meta-Analysis of Outcomes Compared between Robotic and Laparoscopic Gastrectomy for Gastric Cancer. Asian Pac. J. Cancer Prev..

[135] Liu M., Xing J., Xu K., Yuan P., Cui M., Zhang C., Yang H., Yao Z., Zhang N., Tan F. (2020). Application of Near-Infrared Fluorescence Imaging with Indocyanine Green in Totally Laparoscopic Distal Gastrectomy. J. Gastric Cancer.

[136] Li Z., Zhou W., Yang W., Miao Y., Zhang Y., Duan L., Niu L., Chen J., Fan A., Xie Q. (2024). Efficacy and Safety of Robotic vs. Laparoscopic Gastrectomy for Patients with Gastric Cancer: Systematic Review and Meta-Analysis. Int. J. Surg..

[137] Jia Z., Cao S., Wang D., Tang C., Tan X., Liu S., Liu X., Li Z., Tian Y., Niu Z. (2024). Identification and Categorization of Technical Errors and Hazard-Zones of Robotic versus Laparoscopic Total Gastrectomy for Gastric Cancer: A Single Center Prospective Randomized Controlled Study. Ann. Surg..

[138] Suda K., Sakai M., Obama K., Yoda Y., Shibasaki S., Tanaka T., Nakauchi M., Hisamori S., Nishigori T., Igarashi A. (2022). Three-Year Outcomes of Robotic Gastrectomy versus Laparoscopic Gastrectomy for the Treatment of Clinical Stage I/II Gastric Cancer: A Multi-Institutional Retrospective Comparative Study. Surg. Endosc..

[139] Obama K., Kim Y.M., Kang D.R., Son T., Kim H.I., Noh S.H., Hyung W.J. (2018). Long-Term Oncologic Outcomes of Robotic Gastrectomy for Gastric Cancer Compared with Laparoscopic Gastrectomy. Gastric Cancer.

[140] Gao Y., Xi H., Qiao Z., Li J., Zhang K., Xie T., Shen W., Cui J., Wei B., Chen L. (2019). Comparison of Robotic- and Laparoscopic-Assisted Gastrectomy in Advanced Gastric Cancer: Updated Short- and Long-Term Results. Surg. Endosc..

[141] Nakauchi M., Suda K., Shibasaki S., Nakamura K., Kadoya S., Kikuchi K., Inaba K., Uyama I. (2021). Prognostic Factors of Minimally Invasive Surgery for Gastric Cancer: Does Robotic Gastrectomy Bring Oncological Benefit?. World J. Gastroenterol..

[142] Huang K.H., Lan Y.T., Fang W.L., Chen J.H., Lo S.S., Li A.F.Y., Chiou S.H., Wu C.W., Shyr Y.M. (2014). Comparison of the Operative Outcomes and Learning Curves between Laparoscopic and Robotic Gastrectomy for Gastric Cancer. PLoS ONE.

[143] Shibasaki S., Suda K., Kadoya S., Ishida Y., Nakauchi M., Nakamura K., Akimoto S., Tanaka T., Kikuchi K., Inaba K. (2022). The Safe Performance of Robotic Gastrectomy by Second-Generation Surgeons Meeting the Operating Surgeon’s Criteria in the Japan Society for Endoscopic Surgery Guidelines. Asian J. Endosc. Surg..

[144] Suda K., Nakauchi M., Inaba K., Ishida Y., Uyama I. (2016). Minimally Invasive Surgery for Upper Gastrointestinal Cancer: Our Experience and Review of the Literature. World J. Gastroenterol..

[145] Zhou J., Shi Y., Qian F., Tang B., Hao Y., Zhao Y., Yu P. (2015). Cumulative Summation Analysis of Learning Curve for Robot-Assisted Gastrectomy in Gastric Cancer. J. Surg. Oncol..

[146] Huang Q.Z., Wang P.C., Chen Y.X., Lin S., Ye K. (2023). Comparison of Proximal Gastrectomy with Double-Flap Technique and Double-Tract Reconstruction for Proximal Early Gastric Cancer: A Meta-Analysis. Updates Surg..

[147] Sasako M., Sano T., Yamamoto S., Kurokawa Y., Nashimoto A., Kurita A., Hiratsuka M., Tsujinaka T., Kinoshita T., Arai K. (2008). D2 Lymphadenectomy Alone or with Para-Aortic Nodal Dissection for Gastric Cancer. N. Engl. J. Med..

[148] Songun I., Putter H., Kranenbarg E.M.K., Sasako M., van de Velde C.J.H. (2010). Surgical Treatment of Gastric Cancer: 15-Year Follow-up Results of the Randomised Nationwide Dutch D1D2 Trial. Lancet Oncol..

[149] Herrera-Almario G., Patane M., Sarkaria I., Strong V.E. (2016). Initial Report of Near-Infrared Fluorescence Imaging as an Intraoperative Adjunct for Lymph Node Harvesting during Robot-Assisted Laparoscopic Gastrectomy. J. Surg. Oncol..

[150] Lan Y.T., Huang K.H., Chen P.H., Liu C.A., Lo S.S., Wu C.W., Shyr Y.M., Fang W.L. (2017). A Pilot Study of Lymph Node Mapping with Indocyanine Green in Robotic Gastrectomy for Gastric Cancer. SAGE Open Med..

[151] Chen Q.Y., Xie J.W., Zhong Q., Wang J.B., Lin J.X., Lu J., Cao L.L., Lin M., Tu R.H., Huang Z.N. (2020). Safety and Efficacy of Indocyanine Green Tracer-Guided Lymph Node Dissection During Laparoscopic Radical Gastrectomy in Patients with Gastric Cancer: A Randomized Clinical Trial. JAMA Surg..

[152] Baiocchi G.L., Molfino S., Molteni B., Quarti L., Arcangeli G., Manenti S., Arru L., Botticini M., Gheza F. (2020). Fluorescence-Guided Lymphadenectomy in Gastric Cancer: A Prospective Western Series. Updates Surg..

[153] Jeon C.H., Kim S.J., Lee H.H., Song K.Y., Seo H.S. (2023). Indocyanine Green (ICG) in Robotic Gastrectomy: A Retrospective Review of Lymphadenectomy Outcomes for Gastric Cancer. Cancers.

[154] Kim K.Y., Hwang J., Park S.H., Cho M., Kim Y.M., Kim H.I., Hyung W.J. (2024). Superior Lymph Node Harvest by Fluorescent Lymphography during Minimally Invasive Gastrectomy for Gastric Cancer Patients with High Body Mass Index. Gastric Cancer.

[155] Zhang Z., Deng C., Guo Z., Liu Y., Qi H., Li X. (2023). Safety and Efficacy of Indocyanine Green Near-Infrared Fluorescent Imaging-Guided Lymph Node Dissection during Robotic Gastrectomy for Gastric Cancer: A Systematic Review and Meta-Analysis. Minim. Invasive Ther. Allied Technol..

[156] Osterkamp J., Strandby R., Nerup N., Svendsen M.B., Svendsen L.B., Achiam M. (2023). Intraoperative Near-Infrared Lymphography with Indocyanine Green May Aid Lymph Node Dissection during Robot-Assisted Resection of Gastroesophageal Junction Cancer. Surg. Endosc..

[157] Chen Q.Y., Zhong Q., Liu Z.Y., Li P., Lin G.T., Zheng Q.L., Wang J.B., Lin J.X., Lu J., Cao L.L. (2023). Indocyanine Green Fluorescence Imaging-Guided versus Conventional Laparoscopic Lymphadenectomy for Gastric Cancer: Long-Term Outcomes of a Phase 3 Randomised Clinical Trial. Nat. Commun..

[158] Ekman M., Girnyi S., Marano L., Roviello F., Chand M., Diana M., Polom K. (2022). Near-Infrared Fluorescence Image-Guided Surgery in Esophageal and Gastric Cancer Operations. Surg. Innov..

[159] Cousins A., Krishnan S., Krishnan G., Pham N., Milanova V., Nelson M., Shetty A., Ikoma N., Thierry B. (2023). Preclinical Evaluation of Sentinel Node Localization in the Stomach via Mannose-Labelled Magnetic Nanoparticles and Indocyanine Green. Surg. Endosc..

[160] Ikoma N., Badgwell B.D., Mansfield P.F. (2021). Robotic Proximal Gastrectomy with Double-Tract Reconstruction for Gastroesophageal Junction Cancer. J. Gastrointest. Surg..

[161] Nakanishi K., Tanaka C., Kanda M., Shimizu D., Furukawa K., Fujiwara M., Kawashima H., Kodera Y. (2023). Preoperative Indocyanine Green Fluorescence Injection to Accurately Determine a Proximal Margin during Robotic Distal Gastrectomy. Asian J. Endosc. Surg..

[162] Kim M., Son S.Y., Cui L.H., Shin H.J., Hur H., Han S.U. (2017). Real-Time Vessel Navigation Using Indocyanine Green Fluorescence during Robotic or Laparoscopic Gastrectomy for Gastric Cancer. J. Gastric Cancer.

[163] Lee J.H., Son T., Chung Y.E., Cho M., Kim Y.M., Kwon I.G., Kim H.I., Hyung W.J. (2021). Real-Time Identification of Aberrant Left Hepatic Arterial Territories Using near-Infrared Fluorescence with Indocyanine Green during Gastrectomy for Gastric Cancer. Surg. Endosc..

[164] Ito S., Sagawa H., Yamamoto S., Saito M., Ueno S., Hayakawa S., Okubo T., Saito K., Tanaka T., Morimoto M. (2023). Simultaneous Robotic Distal Gastrectomy and Distal Pancreatectomy: Avoiding Total Gastrectomy Using Indocyanine Green Fluorescence Imaging. Asian J. Endosc. Surg..

[165] Kim M.C., Kim W., Kim H.H., Ryu S.W., Ryu S.Y., Song K.Y., Lee H.J., Cho G.S., Han S.U., Hyung W.J. (2008). Risk Factors Associated with Complication Following Laparoscopy-Assisted Gastrectomy for Gastric Cancer: A Large-Scale Korean Multicenter Study. Ann. Surg. Oncol..

[166] Kim K.M., An J.Y., Kim H.I., Cheong J.H., Hyung W.J., Noh S.H. (2012). Major Early Complications Following Open, Laparoscopic and Robotic Gastrectomy. Br. J. Surg..

[167] Hayakawa S., Ogawa R., Ueno S., Ito S., Okubo T., Sagawa H., Tanaka T., Takahashi H., Matsuo Y., Mitsui A. (2022). Impact of the Indocyanine Green Fluorescence Method for Anastomotic Blood Flow in Robotic Distal Gastrectomy. Surg. Today.

